# Genome-wide characterization of *PEBP* family genes in nine *Rosaceae* tree species and their expression analysis in *P. mume*

**DOI:** 10.1186/s12862-021-01762-4

**Published:** 2021-02-23

**Authors:** Man Zhang, Ping Li, Xiaolan Yan, Jia Wang, Tangren Cheng, Qixiang Zhang

**Affiliations:** 1 Beijing Key Laboratory of Ornamental Plants Germplasm Innovation & Molecular Breeding, National Engineering Research Center for Floriculture, Beijing Laboratory of Urban and Rural Ecological Environment, Key Laboratory of Genetics and Breeding in Forest Trees and Ornamental Plants of Ministry of Education, School of Landscape Architecture, Beijing Forestry University, Beijing, 100083 China; 2 Beijing Advanced Innovation Center for Tree Breeding by Molecular Design, Beijing Forestry University, Beijing, 100083 China; 3 Mei Germplasm Research Center, Wuhan, 430073 China

**Keywords:** *PEBP* gene family, *Rosaceae* species, Flowering time regulation in perennials, Gene family evolution, Floral bud break

## Abstract

**Background:**

Phosphatidylethanolamine-binding proteins (PEBPs) constitute a common gene family found among animals, plants and microbes. Plant PEBP proteins play an important role in regulating flowering time, plant architecture as well as seed dormancy. Though *PEBP* family genes have been well studied in *Arabidopsis* and other model species, less is known about these genes in perennial trees.

**Results:**

To understand the evolution of *PEBP* genes and their functional roles in flowering control, we identified 56 *PEBP* members belonging to three gene clades (*MFT*-like, *FT*-like, and *TFL1*-like) and five lineages (*FT*, *BFT*, *CEN*, *TFL1*, and *MFT*) across nine *Rosaceae* perennial species. Structural analysis revealed highly conserved gene structure and protein motifs among *Rosaceae* PEBP proteins. Codon usage analysis showed slightly biased codon usage across five gene lineages. With selection pressure analysis, we detected strong purifying selection constraining divergence within most lineages, while positive selection driving the divergence of *FT*-like and *TFL1*-like genes from the *MFT*-like gene clade. Spatial and temporal expression analyses revealed the essential role of *FT* in regulating floral bud breaking and blooming in *P. mume*. By employing a weighted gene co-expression network approach, we inferred a putative *FT* regulatory module required for dormancy release and blooming in *P. mume*.

**Conclusions:**

We have characterized the *PEBP* family genes in nine *Rosaceae* species and examined their phylogeny, genomic syntenic relationship, duplication pattern, and expression profiles during flowering process. These results revealed the evolutionary history of *PEBP* genes and their functions in regulating floral bud development and blooming among *Rosaceae* tree species.

## Background

Proper timing of flowering is a key adaptive strategy in plant species, especially temperate woody perennials [1–3]. The flowering time in annual or biennials is largely determined by the timing of the transition from vegetative growth to reproductive growth [4, 5]. However, in temperate tree species, flower buds initiate and develop during summer, undergo a short period of dormancy, exit dormancy after exposure to chilling temperatures and finally bloom in suitable environments [6]. Therefore, the blooming time of temperate woody perennials is mainly determined by intrinsic state of flower buds and external environment [7, 8]. Within the context of global climate change, warm winters and irregular occurrences of extreme weather have disrupted the timing of spring phenological events in tree species, increased the risk of frost damage, and caused abnormal fertility and poor fruit setting due to insufficient winter chill [9–12]. Therefore, it is important to study the flowering time control in perennial species and understand their adaptation mechanisms in synchronizing the timing of floral bud breaking and reproduction with local climate [10, 13, 14].

Phosphatidylethanolamine-binding proteins (PEBPs) form a superfamily of genes containing a PEBP domain, which is highly conserved across taxa, from bacteria and insects to mammals and plants [15–17]. Mammalian PEBPs are globular proteins composed of a functional binding site for acetate, phosphate groups and phosphorylethanolamine [18, 19]. Plant PEBP homologs share similar conserved motifs, except their C-terminal part is deleted [20, 21]. Animal PEBP proteins were reported to function as serine proteases or Raf kinase inhibitors, controlling cell growth and differentiation [22–25]. In plants, *PEBP* genes are central regulators in determining the flowering time, plant architecture and seed germination [26–30]. In angiosperms, members of the *PEBP* family fall into three clades of genes: *FLOWERING LOCUS T* (*FT*), *TERMINAL FLOWER 1* (*TFL1*) and *MOTHER OF FT AND TFL1* (*MFT*) [31, 32]. It was reported that *MFT*-like genes exist in both basal land plants and seed plants, while *FT-*like and *TFL1*-like genes were only found in gymnosperms and angiosperms, indicating that the *MFT* clade might be the evolutionary ancestor to *FT-*like and *TFL1*-like genes [32, 33]. Despite extensive sequence similarity among *PEBP* members, their functions have diverged from each other [34].

FT and TFL1 are two major PEBP proteins that are well studied in *Arabidopsis* and in many other plant species [35–38]. In *Arabidopsis*, FT acts as a floral signal transducer, moving from leaves to the shoot apical meristem to promote flowering, while TFL1 maintains inflorescence meristem identity in shoot apex by antagonizing FT functions [39–41]. The balance of *FT* and *TFL1* modulates floral transition and inflorescence architecture by affecting determinacy of meristem identity [30, 42]. FT and TFL1 share ∼ 60% of their amino acid sequence identity, but only a few amino acid changes can convert FT from a floral promoter to a TFL1-like floral repressor [37, 43]. In addition to *FT* and *TFL1*, the *Arabidopsis* PEBP gene family includes *MOTHER OF FT AND TFL1* (*MFT*), *TWIN SISTER OF FT* (*TSF*), *BROTHER OF FT AND TFL1* (*BFT*), and *CENTRORADIALIS* (*CEN*) [27]. MFT integrates abscisic acid (ABA) and gibberellic acid (GA) signaling pathways and acts in a PIF1-dependent manner to repress seed germination under far-red light [28, 44]. *TSF* encodes the closest homolog of FT and resembles FT as a floral inducer under non-inductive SD conditions [45]. BFT and CEN are two floral repressors in *Arabidopsis*, and the overexpression of either one resulted in a late flowering phenotype similar to plants overexpressing *TFL1* [46–48].

Although the PEBP gene family has been recognized as key floral regulators in model species, their molecular evolution and function remains less clear in woody perennials. The *Rosaceae* family consists of over 2500 species from approximately 90 genera, most of which are native to temperate zones around the world [49–51]. *Prunus* is a large genus belonging to the tribe *Amygdaleae* and contains about 430 species, many of which are important fruit crops, such as plums, cherries, apricots and peaches [52]. Additionally, *Prunus* includes a large number of spring-blooming trees with high ornamental and economic value. *Prunus mume* is one of the earliest flowering species, which blooms in late winter or early spring, followed by apricots, peaches, cherries and plums that flower during March to April. Apple and pear trees from the tribe *Maleae* bloom much later, around April to May in Northern China [53]. With the divergent flowering times among *Rosaceae* tree species, it is of great interest to investigate the evolution of *PEBP* family genes and their functional roles in governing flowering time among *Rosaceae* tree species.

Here, we provide a systematic study on the molecular evolution and function of the *PEBP* gene family in *Rosaceae* tree species. We identified 56 PEBP family genes across nine *Rosaceae* species and analyzed the sequence conservation, protein motifs, gene structures, and codon usage patterns of these genes. We then performed genome synteny and duplication analysis, along with nonsynonymous/synonymous substitution (dN/dS) ratio tests, to determine the evolutionary trajectory of *PEBP* family genes. We also analyzed the spatial and temporal expression patterns of *PEBPs* across tissues and in floral buds from floral initiation to bud blooming. Furthermore, we performed weighted gene co-expression network analysis (WGCNA) to determine the *FT* coexpressed genes in *P. mume*. In summary, our study provides insight into the molecular evolution of *PEBP* genes among *Rosaceae* tree species and adds information regarding their function in regulating floral bud development and blooming in woody perennials.

## Results

### Characterization of *PEBP* genes in *Rosaceae* species

**Fig. 1:**
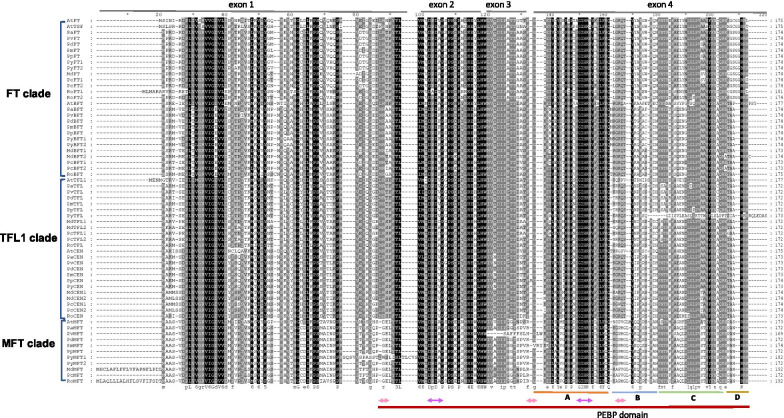
Sequence alignment of 62 PEBP family proteins from nine *Rosaceae* species and *A. thaliana*. The sequences were aligned using Muscle. The conserved protein motif 14-3-3 interaction interface and anion-binding site are underlined in pink and purple, respectively [29]. A, B, C, and D represent four segments in exon 4 [35], which are underlined in orange, blue, green and brown

By combining HMM and BLAST searches, we identified 56 PEBP-like proteins across nine *Rosaceae* tree species (Table 1). Each putative gene was validated by blasting against SMART, Pfam and NCBI CDD to ensure that they contained complete PEBP domain. We then assigned all *Rosaceae* PEBPs to their closest *Arabidopsis* homologs (Fig. 1; Table 1). In total, these *Rosaceae PEBPs* included 12 *FT/TSF*-like, 11 *TFL1*-like, 11 *CEN*-like, 10 *MFT*-like and 12 *BFT*-like genes (Table 1). TFL1 and CEN-like proteins showed the highest identities of 72.25–80.0% with their *Arabidopsis* orthologs, while BFT-like proteins showed the lowest identities of 62.07 to 67.82% compared with AtBFT. Five to six PEBPs were detected among *Prunus* species, while the average number of PEBPs almost doubled in *M. domestica* and *Pyrus communis* (Table 1). The duplicated paralogous gene pairs, such as *MdTFL1* and *MdTFL2*, *PcTFL1* and *PcTFL2*, were retained in the genomes of *M. domestica* and *Pyrus communis*, while only one copy of *MFT* was present in both species (Table 1).

**Table 1:** Detailed information of *PEBP* genes from *A. thaliana* and nine *Rosaceae* species

Gene lineage	Species	Gene accession number	NCBI accession	Notation
*FT*	*Arabidopsis thaliana*	AT1G65480.1; AT4G20370.1		*AtFT*; *AtTSF*
	*Malus domestica*	MD12G1262000	NM_001293862.1	*MdFT*
	*Pyrus communis*	PCP004421.1; PCP023373.1		*PcFT1*; *PcFT2*
	*Rubus occidentalis*	Ro04_G00016; Ro06_G09261		*RoFT1*; *RoFT2*
	*Prunus persica*	Prupe.6G364900.1	XM_007205940.2	*PpFT*
	*Prunus mume*	Pm003733	NM_001293253.1	*PmFT*
	*Prunus armeniaca*	PARG03266m01		*PaFT*
	*Prunus yedoensis*	PQQ05805.1; PQQ09349.1		*PyFT1*; *PyFT2*
	*Prunus avium*	CpS0077204G3m0	XM_021948448.1	*PvFT*
	*Prunus dulcis*	Prudul26A015211P1	XM_034364192.1	*PdFT*
*TFL1*	*Arabidopsis thaliana*	AT5G03840.1		*AtTFL1*
	*Malus domestica*	MD14G1021100; MD12G1023900	NM_001293865.1; NM_001293958.1	*MdTFL1*; *MdTFL2*
	*Pyrus communis*	PCP003730.1; PCP025869.1		*PcTFL1*; *PcTFL2*
	*Rubus occidentalis*	Ro06_G14897		*RoTFL*
	*Prunus persica*	Prupe.7G112600.1	XM_007202602.2	*PpTFL*
	*Prunus mume*	Pm026188	XM_008243028.1	*PmTFL*
	*Prunus armeniaca*	PARG26714m01		*PaTFL*
	*Prunus yedoensis*	PQP96161.1		*PyTFL*
	*Prunus avium*	CpS0034G256m0	XM_021954469.1	*PvTFL*
	*Prunus dulcis*	Prudul26A021958P1	XM_034369048.1	*PdTFL*
*CEN*	*Arabidopsis thaliana*	AT2G27550.1		*AtCEN*
	*Malus domestica*	MD11G1163500; MD03G1143000	NM_001294011.1; NM_001293884.1	*MdCEN1*; *MdCEN2*
	*Pyrus communis*	PCP019918.1; PCP022206.1		*PcCEN1*; *PcCEN2*
	*Rubus occidentalis*	Ro03_G20412		*RoCEN*
	*Prunus persica*	Prupe.6G128400.1	XM_007205944.2	*PpCEN*
	*Prunus mume*	Pm001309	XM_008230265.2	*PmCEN*
	*Prunus armeniaca*	PARG01261m01		*PaCEN*
	*Prunus yedoensis*	PQQ12971.1		*PyCEN*
	*Prunus avium*	CpS00116G158m0	XM_021966077.1	*PvCEN*
	*Prunus dulcis*	Prudul26A027558P1	XM_034364411.1	*PdCEN*
*MFT*	*Arabidopsis thaliana*	AT1G18100.1		*AtMFT*
	*Malus domestica*	MD06G1229900	XM_008376608.2	*MdMFT*
	*Pyrus communis*	PCP033759.1		*PcMFT*
	*Rubus occidentalis*	Ro05_G03590		*RoMFT*
	*Prunus persica*	Prupe.5G230900.1	XM_007209625.2	*PpMFT*
	*Prunus mume*	Pm025099	XM_008241952.1	*PmMFT*
	*Prunus armeniaca*	PARG25179m01		*PaMFT*
	*Prunus yedoensis*	PQQ05244.1; PQQ15508.1		*PyMFT1*; *PyMFT2*
	*Prunus avium*	CpS0021G127m0	XM_021947485.1	*PvMFT*
	*Prunus dulcis*	Prudul26A015523P1	XM_034360958.1	*PdMFT*
*BFT*	*Arabidopsis thaliana*	AT5G62040.1		*AtBFT*
	*Malus domestica*	MD01G1198400; MD07G1265900	NM_001293841.1; XM_008378317.3	*MdBFT1*; *MdBFT2*
	*Pyrus communis*	PCP030682.1; PCP007692.1		*PcBFT1*; *PcBFT2*
	*Rubus occidentalis*	Ro07_G09463		*RoBFT*
	*Prunus persica*	Prupe.2G291900.1	XM_007221111.2	*PpBFT*
	*Prunus mume*	Pm019359	XM_008236052.2	*PmBFT*
	*Prunus armeniaca*	PARG19444m01		*PaBFT*
	*Prunus yedoensis*	PQQ01551.1; PQM34355.1		*PyBFT1*; *PyBFT2*
	*Prunus avium*	CpS0033G388m0	XM_021971342.1	*PvBFT*
	*Prunus dulcis*	Prudul26A027512P1	XM_034348832.1	*PdBFT*

*Rosaceae* species include *M. domestica*, *Pyrus communis*, *R. occidentalis*, *P. persica*, *P. mume*, *P. armeniaca*, *P. yedoensis*, *P. avium* and *P. dulcis*. Gene notations were assigned to *Rosaceae PEBPs* based on their *Arabidopsis* ortholog

### Phylogenetic analyses

**Fig. 2:**
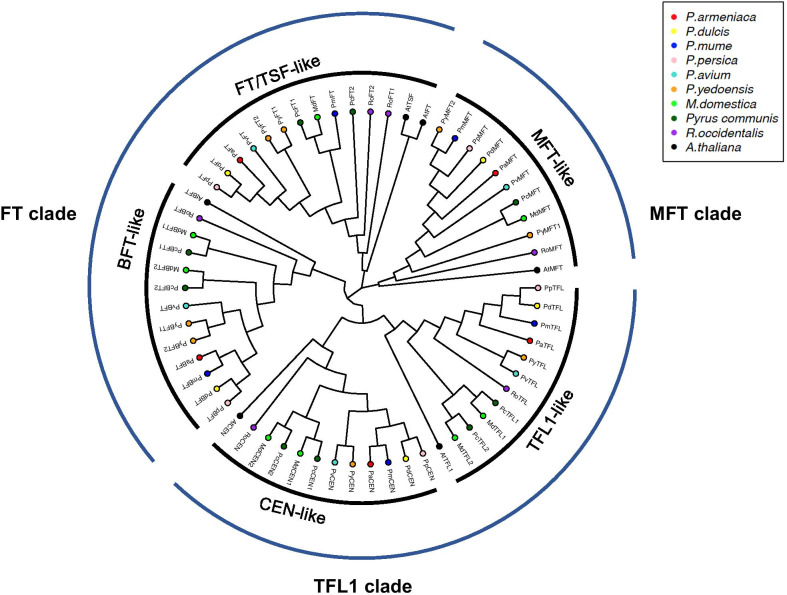
Phylogenetic tree of PEBPs from *Rosaceae* species and *A. thaliana* constructed by the neighbor-joining method. All PEBP proteins can be clustered into three clades and five subfamilies

Phylogenetic trees were constructed based on protein sequence alignment of *Arabidopsis* and *Rosaceae* PEBPs using three approaches: the neighbor-joining, maximum likelihood, and Bayesian inference methods (Fig. 2; Additional file 1: Fig. S1). All three phylogenetic trees shared similar topologies (Fig. 2; Additional file 1: Fig. S1). The phylogenetic trees showed that the 62 PEBP proteins can be clustered into three major clades, which are the *FT*-clade, *TFL1*-clade, and *MFT*-clade (Fig. 2). The *FT*-clade could be further split into *FT/TSF-like* genes and *BFT*-like genes, and the *TFL1*-clade can be split into *TFL1*-like and *CEN-like* subfamily genes (Fig. 2). Within each subfamily, the genes of *Prunus* species first group closely together, then group with the genes of *Maleae* species including *M. domestica* and *Pyrus communis*, and finally group with genes of *R. occidentalis* and *Arabidopsis* (Fig. 2). Among *Prunus* PEBPs, proteins within the same subgenus tend to group together, for example, *P. dulcis* and *P. persica* from the *Amygdalus* subgenus, *P. armeniaca* and *P. mume* from the *Prunus* subgenus, and proteins of *P. yedoensis* and *P. avium* from the *Cerasus* subgenus (Fig. 2). The duplicated paralogous gene pairs from *M. domestica* and *Pyrus communis* within the *TFL1*, *CEN*, and *BFT* subfamilies were grouped separately, for example, *PcTFL1-MdTFL1* and *PcTFL2-MdTFL2* form separate clusters, rather than genes of the same species grouping together (Fig. 2).

### Structural analysis of *PEBP* family genes

**Fig. 3:**
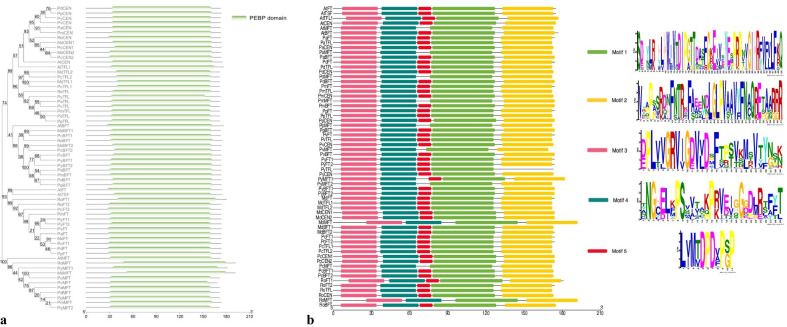
Sequence conservation of the PEBP protein domains within 62 PEBP genes. **a** Phylogenetic analysis of sequences within the PEBP domain; **b** Five major motifs were predicted with MEME and visualized with TBtools

*Rosaceae PEBP* family genes displayed conserved gene structures and high amino acid sequence similarity (Fig. 3; Additional file 2: Fig. S2). The length of the coding regions of *PEBPs* ranged from 507 to 576 bps, with *FT*-like genes falling between 522 to 543 bps, *MFT*-like genes between 507 to 576 bps, *BFT*-like genes between 519 to 525 bps, *TFL1*-like genes between 516 to 519 bps, and *CEN*-like genes between 519 to 522 bps. All *PEBP* genes have a rather loose gene structure consisting of four exons and three introns (Additional file 2: Fig. S2). For example, *BFT*-like genes harbor the shortest intron total lengths, ranging from 522 to 534 bp (Additional file 2: Fig. S2).

Sequence alignment revealed a high degree of conservation across the entire protein and within the PEBP domains present in all 65 genes (Figs. 1, 3). The phylogeny structure inferred from the alignment of PEBP domains was generally in accordance with that of the whole protein sequence alignment, suggesting the PEBP domain as the major factor driving the evolution of *Rosaceae* PEBPs (Fig. 3a). Five motifs covering 160 amino acids were identified by the MEME program among *Rosaceae* PEBP proteins (Fig. 3b). Among these, Motifs 1, 2, 4, and 5 together spread over the whole PEBP domain (Fig. 3b). Motifs DPDXP (Asp-Pro-Asp-X-Pro) and GIHR (Gly-Ile-His-Arg), which are essential for anion-binding activity, were present in the fourth exon of the PEBPs (Fig. 1). We also found that residues distinguishing FT-like from TFL1-like proteins were conserved among the two gene lineages (Additional file 3: Fig. S3). Previously reported key residues conferring the flowering-promoting role of FT including V76, Y91, E115, L134, Y140, G144, W145, Q147, and N159 were present in all *Rosaceae* FT-like proteins (Additional file 3: Fig. S3) [20, 43, 54]. The corresponding residues (I/T)76, H91, E115, (K/N/T)134, (F/N)140, (P/S)144, S145, D147, and D159 were found in all TFL1-like proteins (Additional file 3: Fig. S3). Residues determining the 14-3-3 receptor binding interface (R68, F107, R137) were shared by both protein types (Additional file 3: Fig. S3).

### Microsynteny and duplication analysis of *PEBP* genes

To understand the evolution origin of *PEBP* family genes, we performed inter- and intra-genomic synteny analysis with MCScanX for *Arabidopsis* and seven *Rosacea* species with chromosome-level genome assemblies. We observed large interspecies collinear blocks between four *Prunus* species, *P. avium*, *P. persica*, *P. armeniaca*, and *P. mume*, which indicates high level of macrosynteny among *Prunus* species (Additional file 4: Fig. S4). The genome comparisons between *R. occidentalis* and *M. domestica* and between *M. domestica* and *P. avium* revealed large-scale chromosomal rearrangements including translocation and fusion-fission events that possibly occurred during the genome evolution of *Rubus*, *Malus*, and *Prunus* genera (Additional file 4: Fig. S4). Based on intra-genomic comparisons, we classified the duplication origin of orthologous gene pairs for *Arabidopsis* and other *Rosacea* species (Additional file 11: Table S1). Among all duplication types, whole-genome duplication (WGD)/segmental duplication was the major type for *M. domestica*, tandem duplicated genes were mostly found in *A. thaliana*, *P. armeniaca*, and *P. persica*, and dispersed duplication events were enriched in the genomes of *R. occidentalis*, *P. mume*, *P. avium*, and *P. dulcis* (Additional file 12: Table S1).

**Fig. 4:**
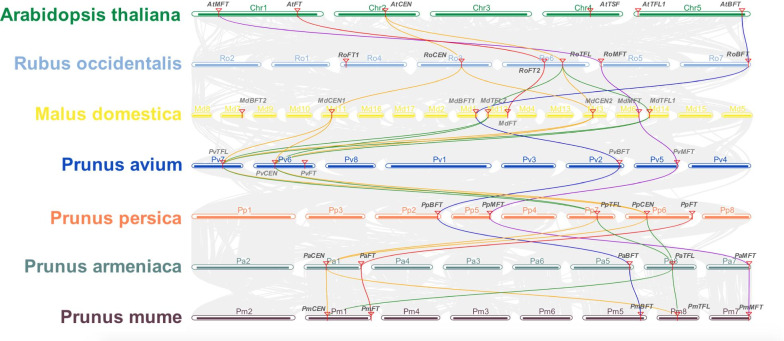
Inter-genomic synteny blocks related to *PEBP* family genes in *A. thaliana*, *R. occidentalis*, *M. domestica*, *P. avium*, *P. persica*, *P. armeniaca* and *P. mume*. Chromosomes of *Rosaceae* species are labeled as Ro, Md, Pv, Pp, Pa, and Pm and are colored differently. We used purple, red, orange, green, and blue lines to connect collinear blocks containing *MFTs*, *FTs*, *CENs*, *TFL1s*, and *BFTs*, respectively

Furthermore, we characterized the duplication modes of *PEBP* family genes across species (Additional file 12: Table S2; Fig. 4; Additional file 5: Fig. S5). In *Arabidopsis*, *R. occidentalis*, and *P. armeniaca*, all *PEBP* gene members were predicted to be originated from dispersed duplications (Additional file 5: Fig. S5; Additional file 12: Table S2). In four *Prunus* species, *FTs*, *MFTs*, and *BFTs* were classified as having dispersed duplication, while *TFL1*-like and *CEN*-like genes were classified as exhibiting WGD/segmental duplication (Additional file 5: Fig. S5; Additional file 12: Table S2). The inter-genomic comparison of *Prunus* species confirmed that *TFL1* and *CEN* genes were within shared syntenic blocks between species, indicating a shared duplication origin of *TFL1*-like and *CEN*-like genes in *Prunus* species (Fig. 4). Within the genome of *M. domestica*, we detected seven syntenic blocks consisting of three WGD/segmental duplication gene pairs, including *MdTFL1*-*MdTFL2*, *MdCEN1*-*MdCEN2*, and *MdBFT1*-*MdBFT2*, and two dispersed duplication events related to *MdFT* and *MdMFT* (Additional file 5: Fig. S5; Additional file 12: Table S2). The inter-genomic comparisons between *M. domestica* and *R. occidental* and between *M. domestica* and *P. avium* also confirmed that the duplicated gene pairs *MdCEN1-MdCEN2, MdTFL1-MdTFL2,* and *MdBFT1*-*MdBFT2* are likely resulted from an independent WGD event unique to the *Malus* tribe (Fig. 4).

### Codon usage bias and other gene parameters

**Fig. 5:**
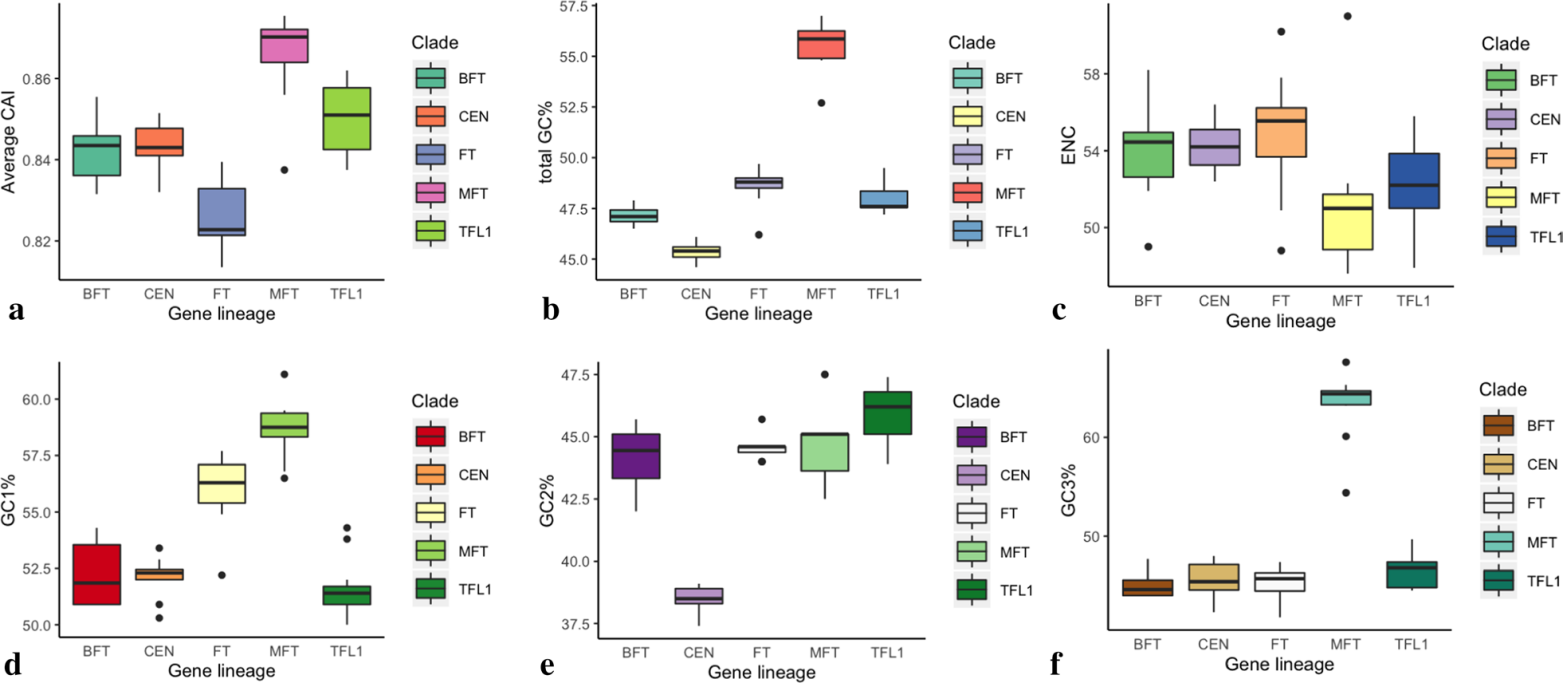
Comparison of gene parameters including the **a** CAI, **b** total GC%, **c** ENC, **d** GC1%, **e** GC2%, and **f** GC3% estimated for *BFT*, *CEN*, *FT*, *MFT*, and *TFL1* genes

We observed differentially preferred codons and different gene features across five *Rosaceae PEBP* gene lineages (Fig. 5; Additional file 6: Fig. S6; Table 2). For arginine, codons AGA and AGG were most frequently used by all lineages compared with other codons (Additional file 6: Fig. S6). Codon UCC encoding serine was mostly used in CEN-like and MFT-like proteins, while codon UCU was mostly employed by the *FT* and *TFL1* lineages (Additional file 6: Fig. S6). We also observed significant differences in other gene parameters among different *PEBP* lineages (all Kruskal–Wallis tests pval < 0.01) (Fig. 5; Table 2). The codon adaptive index (CAI) of *MFT* and *TFL1* genes is significantly larger than those of the other gene groups (Kruskal–Wallis test pval = 2.75e^−7^) (Fig. 5; Table 2). In contrast, the effective number of codons (ENC) estimated for *MFT* and *TFL1* genes is much lower than those of the other groups (Kruskal–Wallis test pval = 0.005) (Fig. 5; Table 2). The average ENC values ranging from 51.24 to 54.95 indicated weak codon bias among *PEBP* genes. Analysis of the GC content revealed that *MFT* lineage genes had much higher GC content indices compared to other genes (Fig. 5; Table 2). In contrast, *TFL1* and *BFT* lineage genes appear to have lower GC1% and GC3% but relatively higher GC2% compared to other groups (Fig. 5; Table 2). All gene parameters showed no variation among species (Additional file 7: Fig. S7; Kruskal–Wallis test pval > 0.05). Strong pairwise correlations between gene parameters were observed (Additional file 13: Table S3). For example, the CAI was positively correlated with the total GC% and GC3% (both correlation coefficient *r* ≥ 0.56), but was negatively correlated with the ENC (*r* = − 0.62) (Additional file 13: Table S3). On the other hand, the ENC displayed a negative correlation with the total GC content and GC3% (Additional file 13: Table S3).

**Table 2:** Average gene parameters estimated for *FT*, *TFL1*, *CEN*, *BFT*, and *MFT* gene lineages

Name	Length	CAI	Total GC%	GC1%	GC2%	GC3%	ENC
*BFT*	524.500	0.842	47.142	52.158	44.133	45.142	54.133
*CEN*	523.364	0.843	45.327	52.118	38.427	45.491	54.264
*FT*	526.750	0.826	48.583	56.058	44.533	45.108	54.950
*MFT*	533.700	0.866	55.500	58.660	44.600	63.240	51.240
*TFL1*	519.273	0.851	47.982	51.627	45.891	46.427	52.291

### Molecular evolution of different *PEBPs* lineages

**Fig. 6:**
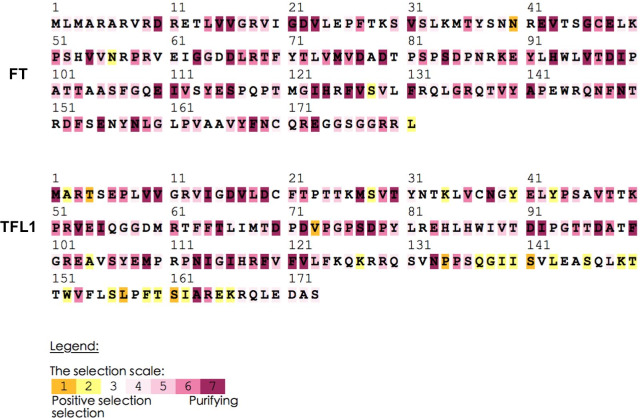
Selective pressure analysis of *FT* and *TFL1* lineage genes identified positively selected sites. Amino acids colored with yellow/purple indicate sites of positive/purifying selection, respectively

To investigate the evolution of *PEBP* genes in *Rosaceae* species, we performed selection scans on coding sequences of all *PEBP*s using the branch model, site model, and branch site model in the CODEML program of PAML (Table 3; Additional file 14: Table S4; Additional file 15: Table S5). Branch models with different ω parameters specified for foreground lineages (i.e., *FT*-like, *TFL1*-like, *CEN*-like, and *BFT*-like lineages and *FT/TFL1* clades) were compared with the fixed ratio model (Additional file 14: Table S4). The likelihood ratio tests (LRT) on models specifying individual lineages of *FT*, *TFL1*, *CEN*, and *BFT* genes as the foreground branch showed no significant difference in ω between the foreground and background branch (P > 0.05) (Additional file 14: Table S4). However, the LRT test on the branch model specifying the *FT* and *TFL1* clades as the foreground branch suggested significant divergence among *FT*/*TFL1* and *MFT* clade genes (P < 0.001) (Additional file 14: Table S4). We then applied the site model LRT test and detected signs of positive selection among sites of PEBP proteins (Additional file 15: Table S5). The branch-site LRT tests further revealed strong positive selection within TFL1 lineage and slight positive selection within FT lineages at specific protein sites (Table 3). The Empirical Bayes model suggested modest selection at positions 19 and 106 when FT lineage was set as the foreground branch and at positions 11 and 18 when TFL1 lineage was set as the foreground branch (Table 3). We further validated the results by performing selective pressure analysis on five gene lineages separately with the software Selecton. Only the FT and TFL1 lineages showed the signature of positive selection, in which residues 40N, 56N, 128S, and 181L in the FT lineage (with RoFT1 as the reference gene) and 4T, 73V, 134P, 141S, 157L, and 161S in the TFL1 lineage (with PyTFL as the reference gene) were mostly selected (Fig. 6). In contrast, the genes of the other three lineages all showed signs of purifying selection across most sites (Additional file 8: Fig. S8).

**Table 3:** Parameter estimates and likelihood values for branch-site models among sites and lineages of PEBP

Branch-site model	Foreground branch	Estimate of parameters	Model comparison	2*|lnL_1_ − lnL_2_|	df	pLRT	Selected sites
Model A	FT	proportion P = 0.54412, 0.44507, 0.00595, 0.00486	Model A vs Model Aa	3.816	1	0.05	19,106
		background ω = 0.13622, 1.00000, 0.13622, 1.00000					
		foreground ω1 = 0.13622, 1.00000, 26.81509, 26.81509					
	TFL1	proportion P = 0.16400, 0.49980, 0.08306, 0.25314	Model A vs Model Aa	10.381	1	0.0013**	11,18
		background ω = 0.14566, 1.00000, 0.14566, 1.00000					
		foreground ω = 0.14566, 1.00000, 999.00000, 999.00000					
	CEN	proportion P = 0.23775, 0.76225, 0.00000, 0.00000	Model A vs Model Aa	2E-06	1	P > 0.05	–
		background ω = 0.14634, 1.00000, 0.14634, 1.00000					
		foreground ω = 0.14634, 1.00000, 1.00000, 1.00000					
	BFT	proportion P = 0.20425, 0.66599, 0.03046, 0.09930	Model A vs Model Aa	2.878	1	P > 0.05	–
		background ω = 0.12885, 1.00000, 0.12885, 1.00000					
		foreground ω = 0.12885, 1.00000, 7.38132, 7.38132					
	(FT, TFL1, CEN, BFT)	proportion P = 0.23291, 0.72041, 0.01140, 0.03528	Model A vs Model Aa	0.521	1	P > 0.05	–
		background ω = 0.15263, 1.00000, 0.15263, 1.00000					
		foreground ω = 0.15263, 1.00000, 10.76576, 10.76576					

Significant chi-squared comparisons are indicated with * (pLRT < 0.05), ** (pLRT < 0.01), and *** (pLRT < 0.001). Positively selected sites in the foreground lineages were detected by Bayes Empirical Bayes analysis with a probability ≥ 0.7

### *Cis*-acting element analysis of the *FT* promoter

We extracted the 2000 bp region of *FT* genes and scanned for putative *cis*-elements by searching against the PlanPan and the PlantCARE databases (Table 4). We compared the type and copy number of cis-elements for 11 *FT* genes from *A. thaliana*, *P. trichocarpa*, *M. domestica*, *Pyrus communis*, *R. occidentalis*, *P. armeniaca*, *P. avium*, *P. mume*, and *P. persica* (Table 4). Within the promoter region of the investigated *FTs*, three to ten CCACA boxes (binding site for CO) were identified across nine species, while none were found for *PtFT2* (Table 4). CArG boxes, the binding site for the MADS-box transcription factor, were present in all *FT* promoters, among which the *AtFT* promoter contained the most (Table 4). Light-response elements including the G-box, AE-box, GATA-motif, GT1-motif, and TCT-motif were present within all *FT* genes but in different types (Table 4). In addition, binding sites for MYB, MYC transcription factor, ethylene-responsive transcription factor, and abscisic-acid responsive element (ABRE) were present in all *FT* promoters (Table 4). Gibberellin-responsive elements of different types were detected in *FT* promoters, with GARE-motif in the promoters of *MdFT*, *PcFT1*, *RoFT1*, *PvFT* and P-box in the promoters of *AtFT*, *PtFT2*, *PaFT*, *PvFT*, *PmFT*, and *PpFT*. We also observed some cis-elements with species-specific distribution patterns. For example, the low-temperature responsiveness (LTR) element was only detected within the promoters of *AtFT*, *PcFT1*, *RoFT1*, *PvFT* and *PmFT* (Table 4). The W-box, which is the binding site for WRKY transcription factor, was detected exclusively in *RoFTs, PtFT2, and Prunus FT* promoter regions (Table 4).

**Table 4:** Summary statistics of putative cis-elements present in the 2 kb upstream promoter region of *FTs* across nine species

TF Family	Organism	Motif	Description	*A. thaliana*	*Populus trichocarpa*		*M. domestica*	*Pyrus communis*	*R. occidentalis*		*P. armeniaca*	*P. avium*	*P. mume*	*P. persica*
				*AtFT*	*PtFT1*	*PtFT2*	*MdFT*	*PcFT1*	*RoFT1*	*RoFT2*	*PaFT*	*PvFT*	*PmFT*	*PpFT*
CCACA box	*A. thaliana*	CCACA	Binding site for CO	5	4	0	6	5	3	10	6	6	6	6
CArG box	*A. thaliana*	CC[A/T]6GG	Binding site for MADS-domain transcription factor	10	1	3	2	2	1	2	2	4	3	5
MYB	*A. thaliana*	CAACAG	Binding site for MYB transcription factor	5	3	1	2	3	4	5	3	3	3	2
bHLH	*A. thaliana*	ATGTG/AGGTG	Binding site for MYC	7	3	3	5	16	8	6	7	6	6	4
TCT-motif	*A. thaliana*	TCTTAC	Part of a light-responsive element	2	2	0	2	1	2	0	4	3	3	3
GATA-motif	*A. thaliana*	AAGATAAGATT	Part of a light-responsive element	0	1	0	1	1	2	2	0	0	0	0
AE-box	*A. thaliana*	AGAAACAA	Part of a module for light response	2	0	1	1	0	0	0	0	0	0	1
G-box	*A. thaliana*	TACGTG	Cis-acting regulatory element involved in light responsiveness	2	1	1	1	2	2	2	3	1	3	2
GT1-motif	*A. thaliana*	GGTTAA	Light-responsive element	2	1	2	0	0	0	2	3	2	3	3
MSA-like		(T/C)C(T/C)AACGG(T/C)(T/C)A	Cis-regulatory element involved in cell cycle regulation	0	0	0	2	0	0	0	0	0	0	0
GARE-motif	*Brassica oleracea*	TCTGTTG	Gibberellin-responsive element	0	0	0	1	1	1	0	0	1	0	0
P-box	*Oryza sativa*	CCTTTTG	Gibberellin-responsive element	1	0	1	0	0	0	0	1	1	1	1
AP2; ERF	*A. thaliana*	CCGAC	Ethylene-responsive transcription factor	7	3	5	8	6	8	6	2	4	2	4
ABRE	*A. thaliana*	ACGTG	Cis-acting element involved in the abscisic acid responsiveness	1	3	2	1	4	4	5	2	1	2	1
LTR	*Hordeum vulgare*	CCGAAA	Cis-acting element involved in low-temperature responsiveness	1	0	0	0	1	1	0	0	1	1	0
W box	*A. thaliana*	CCGAAA	Binding site for WRKY transcription factor	0	0	2	0	0	1	1	1	1	1	1

The organism column indicates in which organism the motif was characterized

### Tissue-specific expression patterns of *PEBPs*

**Fig. 7:**
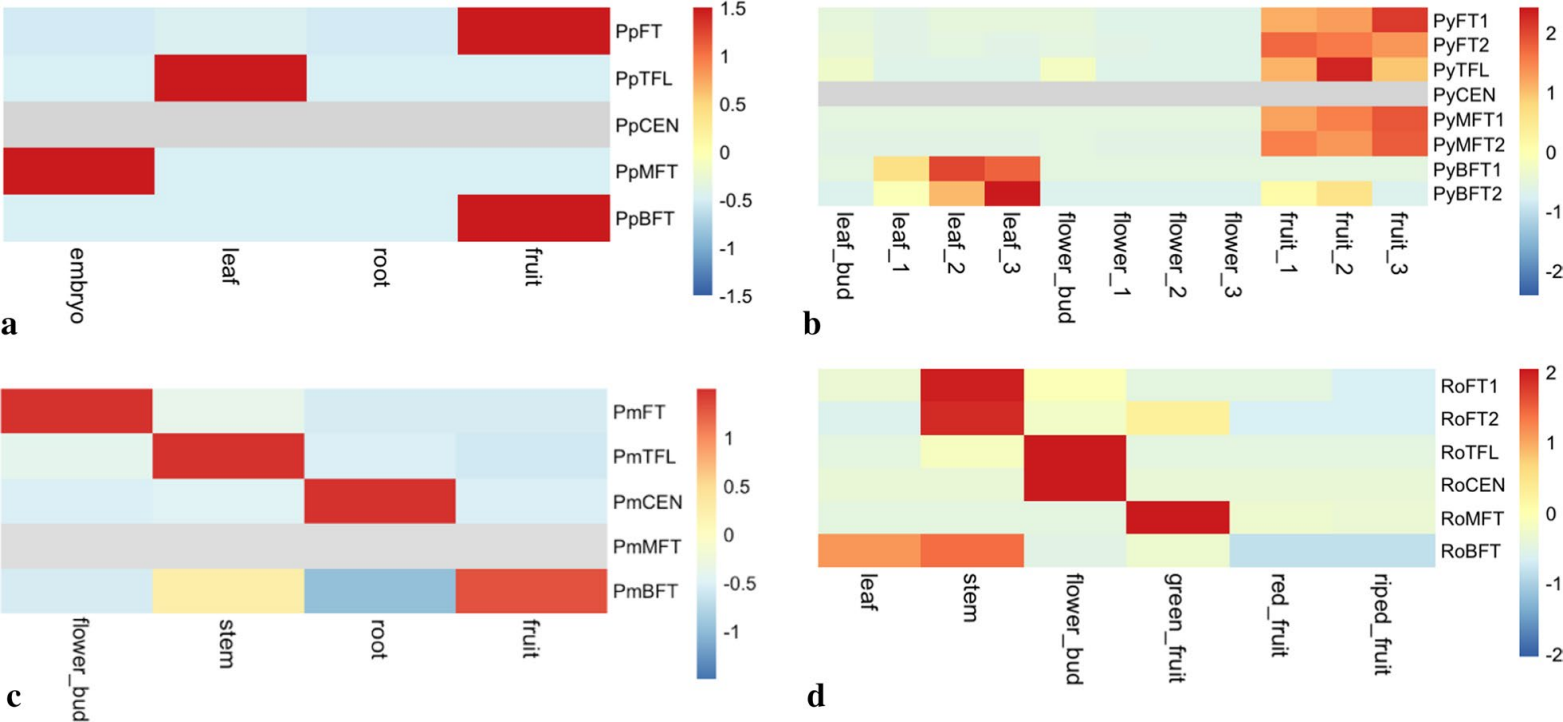
Tissue-specific expression of *PEBP* genes in four *Rosaceae* species including **a** *P. persica*, **b** *P. yedoensis,* **c** *P. mume* and **d** *Rubus occidentalis*

To explore the functional roles of *PEBP* genes, we examined their expression patterns in different tissues of four *Rosaceae* species, *P. persica*, *P. mume*, *P. yedoensis*, and *R. occidentalis* (Fig. 7a–d). In general, we observed a differentiated expression preference of *PEBP* genes across different tissues (Fig. 7). Among the five *PEBP* subfamilie*s*, *FT*-like and *TFL1*-like genes were expressed in both vegetative tissues such as leaf and stem, and reproductive organs such as flower bud and fruit (Fig. 7). The transcription of *CENs*, as the closest paralogs of *TFL1*, was barely detected in any organs, except in the root tissues of *P. mume* (Fig. 7). *MFT* was only detected in seed embryos of *P. persica* and fruit tissues of *P. yedoensis* and *R. occidentalis* (Fig. 7). *BFT* was detected in the fruit tissues of all species but was relatively highly expressed in leaf and stem tissues in *P. yedoensis* and *R. occidentals*, respectively (Fig. 7). We validated the tissue-specific expressions of five *PEBP* genes by real-time quantitative PCR (qRT-PCR) in *P. mume* (Additional file 9: Fig. S9). *PmFT* is highly expressed in floral buds compared with its expression in leaf and stem, which is consistent with result of the above tissue transcriptome sequencing in *P. mume* (Fig. 7; Additional file 9: Fig. S9). *PmTFL* and *PmCEN* were relatively highly expressed in root tissues (Additional file 9: Fig. S9). *PmBFT* and *PmMFT* was barely detected in the four examined tissue types (Additional file 9: Fig. S9). The somewhat inconsistent tissue-specific expression patterns of PEBP orthologs across examined species are likely a result of non-uniformity in the sampling time, plant physiological state, and tissue specificity across four independent studies. Despite the inconsistency, the divergent expression of *PEBP* members across different tissue types indicates significant functional differentiation of *PEBP* gene lineages.

### Expression analysis of *PEBP* genes during floral bud development in *P. mume*

**Fig. 8:**
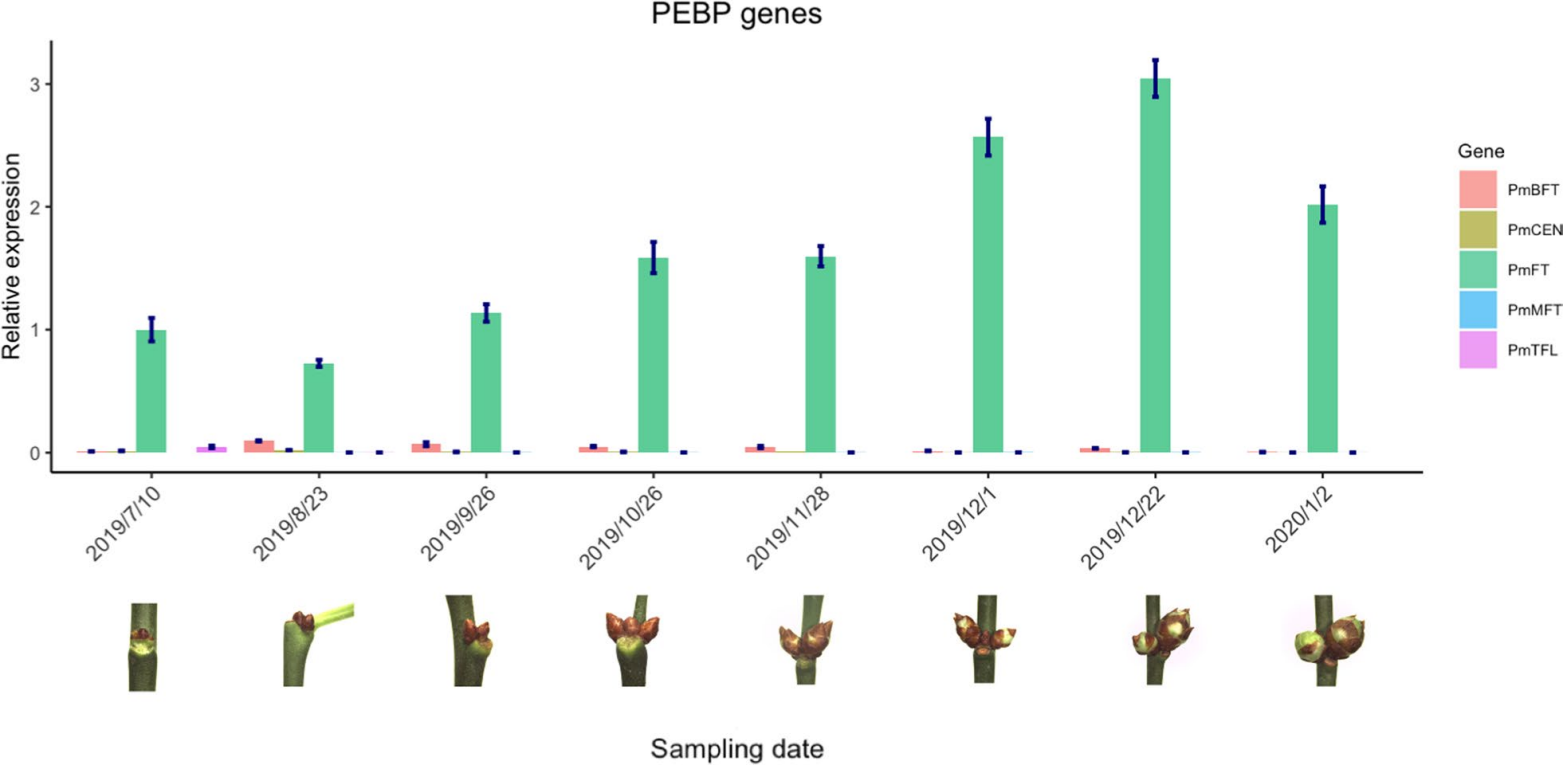
Relative expression levels of *PEBP* genes during floral bud development and bud blooming in *P. mume*

We analyzed the expression of *PEBP* genes in flower buds of different developmental stages from July 10th, 2019 to January 12th, 2020 by qRT-PCR analysis. The expression of *PmFT* first decreased as the bud initiated the floral meristem from July to August, increased as floral organ initiated and developed (from August to October), slightly decreased during bud dormancy, and then significant increased as the floral bud exited dormancy and bloomed (Fig. 8). *PmBFT* maintained a low expression level throughout the whole process, with only a minor increase during floral bud development in August and September (Fig. 8). The other PEBP members retained barely detected expression levels in floral buds of all developmental stages (Fig. 8). These results imply that *PmFT* is possibly the primary *PEBP* member participating in regulating floral bud development and bud flushing in *P. mume*.

### Co-expression network analysis of *FT* during the blooming process in *P. mume*

**Fig. 9:**
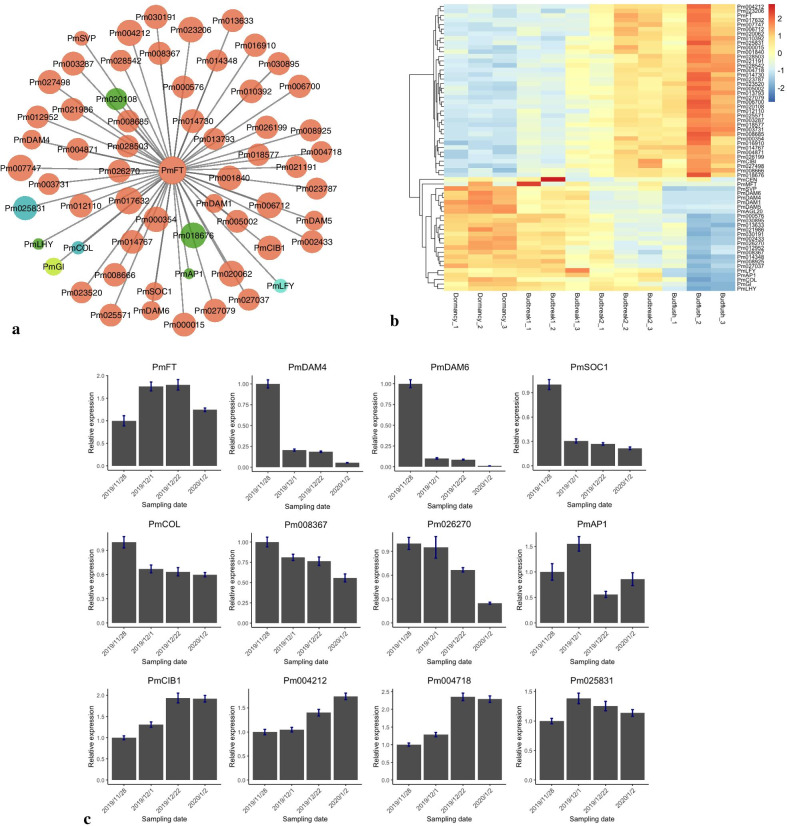
Co-expression network of *FT* during floral bud blooming in *P. mume*. **a** Cytoscape visualization of candidate genes co-expressed with *PmFT* during dormancy release. Candidate genes from the ‘brown’, ‘dark green, ‘green-yellow, ‘turquoise’, and ‘cyan’ modules are colored in brown, green, green-yellow, turquoise, and cyan, respectively. The circle size represents the significance of gene expression correlation with *PmFT*. **b** Expression patterns of *PmFT* and putative co-expressed genes during floral bud blooming. **c** Relative expression of *PmFT* and putative co-expressed genes verified by qRT-PCR analysis

To explore the regulatory network of *FT* in flowering regulation in trees, we reanalyzed the transcriptome changes of *P. mume* during dormancy release and the floral bud opening process [55] and performed a weighted co-expression network analysis (WGCNA). We identified 23 modules with distinct expression patterns (Additional file 10: Fig. S10a). Module-trait association analysis revealed four modules, ‘brown’, 'turquoise', ‘dark green’, and ‘salmon’, associated with the progression of bud flushing (R^2^ > 0.8). Among them, module ‘brown’ showed the strongest correlation with the FPKM of *PmFT* (Additional file 10: Fig. S10b). The ‘brown’ module genes were significantly enriched in biological processes including cell cycle (GO: 0007049), flower development (GO: 0009908), glucan metabolic process (GO: 0009251), auxin transport (GO: 0060918), and responses to abiotic stimulus (GO: 0009628). We further identified the top 50 genes most associated with *PmFT* and 15 known flowering-related genes such as *PmLFY*, *PmAP1*, and *PmCOL* (Additional file 16: Table S6) [56, 57]. Among genes in the ‘brown’ module, *SVP* (*SHORT VEGETATIVE PHASE*), *SOC1* (*SUPPRESSOR OF OVEREXPRESSION OF CO 1*), *GI* (*GIGANTEA*), and *CIB1* (*CRYPTOCHROME-INTERACTING BASIC-HELIX-LOOP-HELIX 1*) were previously identified as key players in the FT-dependent floral regulation in *Arabidopsis* [58, 59] (Fig. 9a). Four tandem-duplicated *PmDAMs* (*PmDAM1*, *PmDAM4*, *PmDAM5*, *PmDAM6*) from the ‘brown’ module also exhibited expression patterns negatively correlated with that of *PmFT* (Fig. 9a, b). The expression patterns of other known floral regulators such as *COL* (*CONSTANS-LIKE*) from the ‘turquoise’, *LHY1* (*LATE ELONGATED HYPOCOTYL 1*) and *AP1* (*APETALA1*) from ‘dark green’ module were not highly correlated with *PmFT* (R^2^ < 0.62) (Fig. 9b). *PmFT* showed a relatively weak transcription level in endodormant floral buds (Fig. 9b). As the floral bud continued accumulating chilling units and exiting dormancy, *PmFT* expression significantly increased and showed the highest expression in flushing buds (Fig. 9b). *PmCIB1* and 37 other genes showed similar expression patterns to that of *PmFT*, while *PmPHYB* (*Pm008367*), *PmGI*, *PmLHY*, *PmCOL*, *PmSVP*, *PmSOC1*, and four *PmDAMs* displayed contrasting expression patterns, with their expression decreasing as the floral buds exited endodormancy (Fig. 9b). The expression patterns of *FT* and its coexpressed genes were further verified by qRT-PCR analysis (Fig. 9c).

## Discussion

### Evolution trajectory of *PEBP* family genes in *Rosaceae* genomes

*PEBPs* form an ancient gene family central to many plant developmental processes, including floral transition, plant architecture, and seed germination [30, 32, 60]. In *Arabidopsis*, the *PEBP* family constitutes six genes grouped into three distinct clades, *FT*-like (*FT* and *TSF*), *TFL1*-like (*TFL1* and *CEN*), and *MFT*-like genes [31]. Though previous studies have characterized the functions of *PEBP* family genes in model plants, none have focused on a comparative analysis of the *PEBP* family in tree species. Our study conducted a systematic search across nine *Rosaceae* genomes and identified 56 *PEBP* family genes orthologous to six *Arabidopsis* genes, *FT/TSF*, *TFL1*, *CEN*, *BFT*, and *MFT*. The number of *PEBP* family members in *Prunus* species (chromosome 2n = 2x = 16) was approximately the same as that in *Arabidopsis* (five to six copies), while *PEBP* members were expanded in *M. domestica* and *Pyrus communis* (chromosome 2n = 2x = 34). Genome synteny and duplication analyses together supported that duplicated ortholog pairs *MdTFL1-MdTFL2*, *MdCEN1-MdCEN2*, and *MdBFT1-MdBFT2* are likely originated from a recent whole-genome duplication (WGD) event that occurred in the *Maleae* clade after splitting from *Prunus* [61]. However, only one copy of *MdFT*, *MdMFT*, and *PcMFT* was retained in apples and pears, indicating that the duplicated copy may have been lost during species evolution after the WGD [62]. The duplication mode analysis also suggested a shared origin of *TFL1* and *CEN* from segmental or WGD duplication in *Prunus* species (Additional file 12: Table S2). Previous studies reported that the angiosperm *TFL1*-like gene experienced duplication after splitting from basal angiosperms, followed by functional divergence, resulting in *TFL1* and *CEN* gene lineages in eudicots [63]. Given the conserved sequence alignment of *Prunus TFL1/CEN* orthologs with other *Rosaceae* species, it is unlikely that *Prunus TFL1/CEN* arose from a recent segmental duplication or WGD unique to *Prunus* species. Therefore, the syntenic relationship may have been caused by the preservation of genomic segments containing *TFL1*, *CEN*, and their neighboring genes through rounds of chromosome rearrangements during *Prunus* species evolution. In *Arabidopsis*, the *TSF* gene, which is a homolog of *FT*, highly resembles *FT* in its coding sequence and flowering promoting role [64]. The absence of *TSF* in the *Rosaceae* genome suggests that the gene duplication of *FT/TSF* possibly occurred in *Brassicaceae* after splitting from their common ancestors [65].

The *PEBP* gene family experienced two ancient duplications, giving rise to three types: *FT*-like genes promoting flowering, *TFL1*-like genes repressing flowering and maintaining indeterminate state of meristems, and *MFT*-like genes controlling seed germination [17, 27, 32]. The phylogenetic analysis suggests that *Rosaceae PEBPs* can be clustered into three distinct clades (*FT*, *TFL1*, and *MFT*), which is consistent with other species [17, 27, 32]. The *FT*-like clade can be further divided into *FT* and *BFT* lineages, and the *TFL1*-like clade can be divided into *TFL1* and *CEN* lineages. Based on maximum-likelihood test on branch models specifying different gene lineages (*FT*, *TFL1*, *CEN*, *MFT*, and *BFT*) as the foreground branch, we detected no evidence of positive selection acting on any of them. However, we observed significant selection acting on *FT*/*TFL1* clade genes with the *MFT* clade specified as the background branch, which supports the theory that functional divergence of the *FT*/*TFL1* clade occurred after splitting from the *MFT* clade [33]. Through likelihood ratio tests on branch-site models, we detected a few slightly selected codons within the *FT* lineage and a few strongly selected codons in the *TFL1* lineage, which is consistent with results of Selecton analysis on individual lineages. In summary, these results indicate that adaptive evolution is driving the divergence of the *FT* and *TFL1* clades from the *MFT* clade, as well as the diversification among *FTs* and *TFL1s* in *Rosaceae* species. These result are consistent with a previous study reporting that positive selection on *FT-like genes* especially within the fourth exon is driving their divergence from *MFT* and *TFL1* clade [17]. We also observed strong purifying selection constraining protein evolution within the *MFT*, *CEN*, and *BFT* lineages in *Rosaceae* species. However, this does not rule out the possibility of positive selection acting on a few codons masked by strong purifying selection in preserving the other sites [17].

Additionally, we examined the codon usage patterns of *PEBP* genes across *Rosaceae* species. Codon usage bias refers to the nonrandom choice of synonymous codons in specific genes or species and can affect the translation efficiency and accuracy, protein folding, and biological functions [66, 67]. The codon usage pattern usually reflects the balanced effect of mutation pressure and selection constraints during long-term evolution [68, 69]. Several codons for amino acids were differentially preferred across five *PEBP* lineages. Among all codons, the most frequently used codon for arginine was AGG for *FT*, *CEN*, and *MFT* and AGA for the *BFT* and *TFL1* lineages (Additional file 6: Fig. S6). Several other codons, including TCC, TCA, TCT for serine and CCT for proline, were preferred by specific *PEBP* gene lineages, indicating differentially selected codons by different *PEBP* gene lineages. To further understand the factors influencing codon usage patterns, we compared the GC content, gene length, CAI, and ENC of different *PEBP* lineages and species. The CAI measures the optimal codon usage for a gene and is commonly used as an index for the expression level [70]. The ENC has been widely used to determine the level of codon bias for individual genes [71]. We observed significant differences in these gene features estimated for different gene lineages but not for species. Despite the differences, all genes had a relatively high CAI (range 0.81–0.87) and moderate ENC (above 47), indicating high translational efficiency and slightly biased codon usage among *PEBP* genes. Furthermore, the strong pairwise correlations between ENC and GC content, ENC and CAI indicate that the nucleotide composition and gene expression level are two factors possibly contributing to the differentiated codon preference among different *PEBP* gene lineages [69].

### Functional role of *FT*/*TFL1* genes in *Rosaceae* tree species

Structural analysis of *Rosaceae* PEBP proteins revealed a highly conserved gene structure and amino acid sequence, especially within the PEBP functional domain (Figs. 1, 3; Additional file 2: Fig. S2). All PEBP family genes shared a common gene structure with exactly four exons of similar sizes. Among the conserved protein motifs, the anion-binding D-P-D-x-P and G-x-H-R motifs are important for the conformation of the ligand binding site in PEBP proteins [72]. Mutations close to this region may affect the binding of FT protein with phosphate ions and thus alter its interaction with FD (FLOWERING LOCUS D) [73]. Segment B on exon 4 encodes an external loop, and together with its adjacent segment C, determines the opposite functions of *FT* and *TFL1* in *Arabidopsis* [35]. Another key protein motif is the 14-3-3 binding domain that is essential for FT/TFL1 interaction with 14-3-3 receptors to modulate flowering [20]. Key residues within these motifs are critical in determining FT/TFL1 functions. For example, the substitution of an amino acid (replacing His-88 in TFL1 with Tyr) can convert TFL1 into a floral promoter [37]. In another study, specific mutations at four residues—Glu-109, Trp-138, Gln-140, and Asn-152—converted FT into a TFL1-like repressor [43]. The amino acids at each of these critical positions were highly conserved and specific to FT-like and TFL1-like proteins, which suggests that the floral promoting and repressing role of *FT*/*TFL1* genes in *Rosaceae* species is possibly conserved.

Recent molecular studies have characterized the function of *Rosaceae FT*/*TFL*-like genes in several *Rosaceae* perennials [33]. The overexpression of *MdFT* in both *Arabidopsis* and apple lead to precocious flowering [74]. The ectopic expression of *PmFT* and *RoFT* in tobacco leads to extremely advanced flowering [75]. Similarly, the late-flowering phenotype of *Arabidopsis ft* mutant can be rescued by overexpressing *PpFT*, indicating the conserved floral promoting role of *FT* in examined *Rosaceae* species [76]. On the other hand, prolonged vegetative growth and a late-flowering phenotype were observed for transgenic *Arabidopsis/tobacco* overexpressing *PpTFL1*, *PmTFL1*, *RoTFL1*, *MdTFL1*-1/2, suggesting that the *Rosaceae TFL1*-like genes can complement the *TFL1* function in *Arabidopsis* [77–79].

Despite the conservative function of *Rosaceae FT*/*TFL1*-like genes in herbaceous plant systems, their regulatory roles in perennial trees may differ. For example, two homologs of *PcFTs* showed differed annual expression patterns in the apical buds of *Pyrus communis* [80]. The ectopic expression of *PcFT2* caused early flowering in tobacco but delayed dormancy and leaf senescence in *M. domestica* [80]. Another study in pears reported that the expression of *FTs* was not induced in the reproductive meristem prior to floral initiation, while the transcripts of *TFL1s* rapidly decreased and maintained a very low level, indicating the essential role of *TFL1* in floral induction in *Pyrus pyrifolia* [36]. In our study, the minimal level of *TFL1* throughout all floral bud stages may indicate that the repression of *TFL1* is necessary for determinate floral meristem identity and terminal flower formation during floral bud development in *P. mume*. The multifaceted role of *FT*/*TFL1*-like genes was also observed in other tree species [33]. In poplar, *PtFT1* functions as a floral promoter activated by chilling temperatures, while vegetative growth and dormancy breaking are promoted by *PtFT2* [81]. Plum trees transformed with *PtFT1* displayed a shrub-like growth habit, a reduced chilling requirement, and insensitivity to short-day signals [82]. In gymnosperms, *FT*-like genes exhibited contrasting roles in regulating growth cycling and bud setting [83]. For example, expression of *FT*/*TFL1*-like genes in Norway spruce (*PaFTL2*) and Scots pine (*PsFTL2*) increase during bud setting in autumn and decrease during bud bursting in the next spring [84–86]. Thus, *FT*/*TFL1*-like genes may undertake some novel functions concerning floral transition, plant architecture, and growth-dormancy cycling during the evolution of tree species.

### Regulatory role of *FT* in promoting bud break and blooming in perennial trees

Flowering is a major developmental process that is key to the fitness and reproduction of higher plants [87]. Plants have synchronized their seasonal timing of flowering with favorable environmental conditions to ensure sexual reproduction success and seed production [87, 88]. The regulation of flowering times requires an intricate network of signaling pathways, which has been studied in many plant species but is best characterized in *Arabidopsis* [57, 87, 89]. *FT* functions as a gene hub integrating five major floral induction pathways, including the photoperiodic pathway, vernalization pathway, autonomous pathway, gibberellin pathway, and age pathway [56, 59]. In *Arabidopsis*, the transcription of *FT* is activated by the transcription factor CONSTANS (CO), which is affected by the circadian regulatory GI [90, 91]. The *GI-CO-FT* module not only is used to regulate photoperiod-dependent flowering in *Arabidopsis* and temperate cereals [92, 93] but also showed a conserved function in regulating short-day induced bud dormancy in poplar [94]. In addition to CO, SVP, FLC (FLOWERING LOCUS C), and PIF4 (PHYTOCHROME INTERACTING FACTOR 4) from the vernalization pathway can also regulate *FT* transcription through directly binding the *FT* promoter or intronic regions [90, 95–97]. Upon induction by long-photoperiod signals, FT, together with other floral pathway integrators SOC1 and LFY (LEAFY), activates floral meristem identity genes such as *AP1*, *APETALA2* (*AP2*), *FRUITFULL* (*FUL*), *CAULIFLOWER* (*CAL*), and *LFY*, which convert the vegetative meristem to floral meristem in *Arabidopsis* [59, 98, 99].

Though flowering regulation is well understood in model species, it is still unclear in temperature tree species. Unlike annual or biennials, many trees in temperate environments initiate floral buds in the preceding summer, cease growth in autumn, with floral buds remaining dormant during winter, and then bloom early in spring after exposure to chilling temperatures [6, 10]. Therefore, perennial flowering marks the event of the floral bud exiting dormancy and flushing instead of the time of floral meristem initiation in annual species [6]. So far, many studies on floral bud breaking regulation have been reported; however, the molecular mechanism is still far from complete. Apart from regulating floral initiation, *FT* has been suggested to participate in regulating bud dormancy in temperate trees [100]. Poplar exhibited constitutive expression of *FT1* initiated flower-like structures directly from tissue culture and showed delayed growth cessation in short-days [81, 94], while *FT2* was predominantly expressed during vegetative growth and is likely responsible for growth cessation and vegetative bud set [81]. Moreover, *Rinne *et al. (2011) reported that *FT* is hyper-induced during bud breaking in poplar, indicating that *FT* may also participate in regulating dormancy release in poplar [101]. In pear, chilling reduces the expression of *DAM* genes, which are well-known floral repressors, releasing the repression of *FT* and promoting floral bud breaking [102, 103]. Our expression analysis confirmed that *FT* is significantly induced during chilling-mediated floral bud breaking in *P. mume*.

To further understand the regulatory module of *FT* during floral bud breaking, we used WGCNA and identified a number of candidate genes whose expression patterns strongly correlated with *FT* in *P. mume*. Among these candidates, *PmDAM1*, *PmDAM4*, *PmDAM5*, and *PmDAM6* were found to be downregulated during the progression of bud breaking. Another MADS-box gene *PmSVP* displaying a similar expression pattern to that of *PmDAMs* was reported to maintain bud dormancy in apples [104]. Thus, PmDAMs and PmSVP may function as *FT* repressors in the same manner as in *Arabidopsis* by binding to the CArG box in the promoter region of *PmFT* [105]. A number of genes previously identified upstream *FT*, including *PmCOL*, *PmGI*, and *PmCIB1*, were found to be induced by chilling in endodormant buds before the activation of *PmFT*. These genes may act directly or indirectly to activate *FT* expression during dormancy release in *P. mume*. We also observed that some known FT regulated genes, namely, *AP1*, *SOC1*, and *LFY*, peaked before the induction of *FT*, indicating their functional role during flower bud development prior to bud breaking [6, 106]. Additionally, a number of *FT* co-expressed genes were annotated to pathways that did not show relatedness to bud breaking or flowering in previous studies. Future functional studies are required to characterize the regulatory mechanisms of *FT* in floral induction and bud breaking in *Rosaceae* tree species.

## Conclusions

In this study, we systemically characterized the *PEBP* gene family in nine *Rosaceae* species and examined their gene structure, protein features, evolutionary trajectories, and expression profiles. The 56 *PEBP* genes can be divided into three major clades, namely, *FT*-like, *TFL1*-like, and *MFT*-like genes. We observed highly conserved protein motifs and gene structure among *PEBP* genes. Selection scans showed that positive selection is driving the divergence of the *FT* and *TFL1* clades, while strong purifying selection is restraining diversification within most lineages. Expression analysis of *PEBP* genes suggested the essential role of *FT* in floral bud development and blooming. Furthermore, we identified a number of *FT* co-expressed genes, revealing a *FT*-related regulatory model in *Prunus* species different from those in annual or biennial plants. In summary, the comprehensive analysis of the *PEBP* family in our study provided evidence of structural and functional conservation of *PEBP* genes among *Rosaceae* woody perennials and provided insight into the adaptive evolution of the *PEBP* gene family over the evolutionary history of perennial trees.

## Methods

### Identification of the *PEBP* gene family

We obtained the most recent versions of genomes for *P. persica* [107], *P. mume* [108], *P. yedoensis* [109], *P. avium* [110], *P. dulcis*, *P. armeniaca* [111], *M. domestica* [112], *Pyrus communis* [113], and *R. occidentalis* [114] from GDR (Genome Database For *Rosaceae*) [115]. To identify the *PEBP* genes of each species, we retrieved the HMM model PF01161 of PBP domain from the Pfam database (https://pfam.xfam.org) and searched the genome protein databases with an e-value cutoff of 1.0 × e^−5^ using HMMER 3.1 software [116]. In addition, we used protein sequences AtFT (At1g65480.1), AtTSF (At4g20370.1), AtTFL1 (At5g03840.1), AtBFT (At5g62040.1), and AtMFT (At1g18100.1) downloaded from TAIR (The Arabidopsis Information Resource) (www.arabidopsis.org) as query sequences to blast against the local protein databases of nine species, and we only retained putative PEBP proteins with identities > 40% and e-values ≤ 1.0 × e^−10^. The genes identified by both methods were considered as candidate PEBP family genes and were then verified with SMART [117], Pfam [118], and the CDD database [119] to ensure the completeness of the PBP domain. Redundant sequences or sequences with incomplete PEBP domain were excluded from the following analyses.

### Phylogenetic analysis

Multiple sequence alignment was performed using the protein sequences with software MUSCLE v3.8 [120] and was visualized with GeneDoc v2.6 [121]. Phylogenetic trees were constructed using neighbor-joining (NJ) method with MEGA7 [122], maximum likelihood (ML) analysis with RAxML v8.1 [123], and Bayesian inference (BI) with MrBayes 3.1 [124]. Bayesian inference was performed with 100,000 generations of Markov-chain Monte Carlo (MCMC) simulations, discarding the first 2500 trees as ‘burn-in’. With consistent tree topologies inferred by these three approaches, the neighbor-joining tree was chosen to display the phylogeny of *Rosaceae* full PEBP protein sequences. Furthermore, amino acids within the regions of predicted PEBP domains were extracted and used to construct a PEBP domain tree by the NJ method.

### Gene structure and protein motif detection

The exon and intron locations of *PEBP* genes were analyzed by comparing the coding sequences with their genome sequences. The MEME (Multiple Expectation Maximization for Motif) online tool (http://meme-suite.org/tools/meme) was used to predict protein motifs [125]. The protein motifs were further annotated with the Pfam [118], SMART [117] and CDD [119] online tools. The chromosome distributions of *PEBP* genes were obtained based on genome GFF3 files. Finally, the gene structures, protein motifs, and chromosome locations were visualized with the software TBtools [126].

### Microsynteny analysis and codon usage evaluation

To identify the synteny of *PEBP* family genes among species, we performed all-to-all BLASTP between the genomes of *A. thaliana*, *R. occidentalis*, *M. domestica*, *P. avium*, *P. persica*, *P. armeniaca*, and *P. mume*. We also performed self-blast by comparing protein-coding genes against their own genome using BLASTP. All BLASTP hits with e-values < 1e^−10^ were used as input for software MCScanX (Multiple Collinearity Scan toolkit) [127] to identify possible collinear blocks within and between genomes of different species. Based on the self-blast results, we classified the duplication origin of orthologous genes pairs including *PEBP* family genes with the ‘duplicate_gene_classifier’ toolkit built in MCScanX for each species. All intra/inter-genomic synteny relationships were visualized with TBtools [126].

Gene parameters including the GC content (total GC%, GC1%, GC2%, and GC3%), CAI, and ENC were computed using CAICal (http://genomes.urv.cat/CAIcal/) [128, 129]. CAI provides an estimate of directional translational selection in optimizing the codon usage patterns of genes and is used to predict highly expressed genes [70]. ENC is a number between 20 to 61 that measures the degree of codon usage bias (where ENC = 20 refers to the preference of only one codon per amino acid, while ENC = 61 refers to complete unbiased codon usage) [130]. We compared these gene parameters for *FT*, *TFL1*, *CEN*, *BFT*, and *MFT* gene lineages and across species using the Kruskal Wallis Test with the ‘kruskal.test’ function in R. The relative synonymous codon usage (RSCU) is defined as the ratio of the observed codon frequency to the expected frequency of all synonymous codons per amino acid and is calculated using software MEGA7 [131].

### Molecular evolution of *PEBP* genes

To investigate the signatures of positive selection on *Rosaceae PEBP* genes, we extracted the coding sequences of PEBP genes and aligned them with MUSCLE v3.8 [120]. The sequence alignment was then trimmed with Gblocks [132] in ‘codon’ mode, and the resulting alignments were used to infer phylogenetic relationships with RAxML [123]. The ratios (*ω*) of nonsynonymous substitution sites (*dN*) and synonymous substitution sites (*dS*) were computed for each PEBP lineage gene using the branch model, site model and branch-site model with the codeml package in PAML 4.0 [133]. To test the hypothesis of adaptive evolution in specific *PEBP* lineages and across sites, we performed likelihood ratio tests to evaluate the fit of branch models (*FT*, *TFL1*, *CEN*, *BFT* and (*FT*, *TFL1*, *CEN*, *BFT*) set as foreground branch), site models, and branch site models. The positively selected sites were detected by Bayes Empirical Bayes analysis in PAML 4.0 [133]. To better visualize the site-specific selection on amino acids within each *PEBP* lineage, we performed a selection pressure test with site model M8 and visualized the results with Selecton Server [134].

### *Cis*-element analysis of the *FT* promoter region

To investigate the conservation of the cis-regulatory model of *FT* genes across different species, we extracted the 2 kb upstream region of the start codon (ATG) and submitted the sequences to the PlantCARE [135] and PlantPan 2.0 databases [136]. The *cis*-acting elements predicted by both methods were integrated and considered as putative *cis*-acting elements.

### Tissue-specific expression profiles of *PEBP* genes

The tissue transcriptome sequencing data of *P. mume*, *P. yedoensis*, *P. persica*, and *R. occidentalis* was retrieved from four independent studies: GSE4760162 from the GEO database [108] and SRP136962, SRA053230, and SRP149938 from the NCBI SRA database [109, 137]. The raw SRA files were first dumped to FASTQ format using SRA toolkit and preprocessed with Trimmomatic v0.38 [138] to trim off poor-quality reads. Clean paired reads were aligned with the reference genomes of *P. mume*, *P. yedoensis*, *P. persica*, and *R. occidentalis*, respectively, with software HISAT2 [139]. The genic count was computed with HTSeq [140] and normalized to RPKM with R package ‘edgeR’ [141]. The RPKM value of each PEBP gene across different tissues of *P. mume*, *P. persica*, *P. yedoensis*, and *R. occidentalis* was extracted and visualized using the ‘*pheatmap*’ package in R. The relative expression of *PEBP* genes in leaf, stem, root, and floral bud tissues was tested in *P. mume* using real-time PCR analysis with detailed procedure described below.

### Expression analysis of *PEBP* genes during the flower bud development process

To further understand the functional role of *PEBP* genes in floral bud initiation and the bud flushing process, we performed real-time quantitative PCR analysis to examine the temporal expression patterns of *PEBP* genes. Lateral floral bud samples were collected from *P. mume* ‘Fei Lve’ tree grown in the Jiufeng sunlight greenhouse approximately every four weeks from July 10th, 2019 to January 12th, 2020. The total RNA was extracted from mixed bud samples using the E.Z.N.A.® Plant RNA Kit following the manufacturer’s instructions (Omega Bio-tek, Norcross) and was reverse-transcribed into cDNA using the PrimeScript RT reagent kit with gDNA Eraser (Takara, Japan). We performed real-time PCR experiments with at least three technical replicates on the PikoReal real-time PCR platform (Thermo Fisher Scientific, Germany). The temperature was set as follows: 95 °C for 30 s; 40 cycles of 95 °C for 5 s, 60 °C for 30 s; 60 °C for 30 s; and ending 20 °C. We used protein phosphatase 2A (PP2A) as an internal reference and calculated the relative transcription levels of target genes using the 2 − ΔΔCt method [142]. The primers used for qRT-PCR experiments are listed (Additional file 17: Table S7).

### Co-expression network of *FT* during the blooming process in *P. mume*

To investigate the functional role of *FT* during floral bud flushing, we obtained the transcriptome data of four successive stages during dormancy release and blooming in *P. mume* from a previous study reported by Zhang et al*.* (2018) [55]. The procedure of sample collection, RNA extraction, sequencing library construction, quality control, and gene expression quantification was described in detail [55]. We normalized the gene expression and performed weighted gene co-expression network analysis with WGCNA v1.67 package in R [143]. The Dynamic Tree Cut algorithm was applied to detect gene modules (power β of 4; height cutoff of 0.3; minimal module size of 30). To identify the key modules coexpressed with *FT*, we calculated the module-trait association and ranked genes by their correlation with the FPKM value of *PmFT*. Finally, the top 50 candidate genes (R^2^ > 0.6) coexpressed with *PmFT* and 15 *FT* interacting factors identified in *Arabidopsis* flowering pathways [56, 57] were selected to construct the coexpression network of *FT*. The *FT* regulatory network was visualized with Cytoscape 3.1 [144]. The expression levels of *FT* and putative co-expressed genes were further validated by qRT-PCR analysis. The primers are described in the supplementary data (Additional file 18: Table S8).

## Supplementary Information

**Additional file1: Fig. S1.** Phylogenetic trees were constructed using three different algorithms.

**Additional file2: Fig. S2.** Exon-intron distributions of *PEBP *family genes from nine *Rosaceae *species and *A. thaliana*.

**Additional file 3: Fig. S3.** Protein sequence alignment of FT and TFL1-like proteins in *Arabidopsis* and nine *Rosaceae* species.

**Additional file4: Fig. S4.** Inter-genomic synteny blocks between species.

**Additional file 5: Fig. S5.** Distribution and collinearity of *PEBP *family genes within genomes of nine *Rosaceae *species.

**Additional file 6: Fig. S6.** Heatmap of average RSCU scores estimated for different codons in five *Rosaceae* *PEBP* gene lineages.

**Additional file 7: Fig. S7.** Comparison of gene parameters including the (a) CAI, (b) total GC%, (c) ENC, (d) GC1%, (e) GC2%, (f) GC3% for all *PEBP* genes across nine *Rosaceae* species.

**Additional file 8: Fig. S8.** Selective pressure analysis of the *BFT*, *CEN*, and* MFT* lineages and *PEBP *genes as a whole identified strong purifying selection across protein sites.

**Additional file 9: Fig. S9.** Relative expression of *PEBP *family genes in floral bud, leaf, stem, and root tissues of *P. mume*.

**Additional file 10: Fig. S10.** Weighted gene co-expression network analysis (WGCNA) during four stages of floral bud blooming in *P. mume*.

**Additional file 11: TableS1.** Numbers of genes originated from different types of duplication events in the genomes of *Arabidopsis* and seven *Rosaceae* species.

**Additional file 12: TableS2.** The duplication modes of *PEBP *genes in the genomes of *Arabidopsis *and seven *Rosaceae* species.

**Additional file 13: TableS3.** Pearson correlation between gene parameters estimated for 56 *Rosaceae* *PEBP* genes.

**Additional file 14: TableS4.** Parameter estimates and likelihood values for branch models among *PEBP* lineages.

**Additional file  15: TableS5.** Parameter estimates and likelihood values for site models across PEBP protein sites.

**Additional file 16: TableS6.** Candidate genes co-expressed with *PmFT*during dormancy release in *P. mume*.

**Additional file 17: TableS7.** Primers used in qRT-PCR analysis for *PEBP*family genes in *P. mume*.

**Additional file 18: TableS8.** Primers used in qRT-PCR analysis for *FT*co-expressed genes in *P. mume*.

## Data Availability

The datasets analyzed during the current study are available from the corresponding author on reasonable request.
