# Supplementary material for: Genome-wide characterization of PEBP family genes in nine Rosaceae tree species and their expression analysis in P. mume
Source: BMC Ecol Evol. 2021 Feb 23;21:32. doi: 10.1186/s12862-021-01762-4 (PMC7901119; doi:10.1186/s12862-021-01762-4)
Supplement: Supplementary file 1 — Additional file1: Fig. S1. Phylogenetic trees were constructed using three different algorithms. [file 12862_2021_1762_MOESM1_ESM.pdf]

Figure S1. Phylogenetic trees were constructed using three different algorithms. (a) NJ (neighbor-joining) tree, (b) ML (maximum likelihood) tree, (c) Bayes (Bayesian) tree. Genes were clustered into *FT*-like, *BFT*-like, *TFL*-like, *CEN*-like, and *MFT*-like subfamilies.

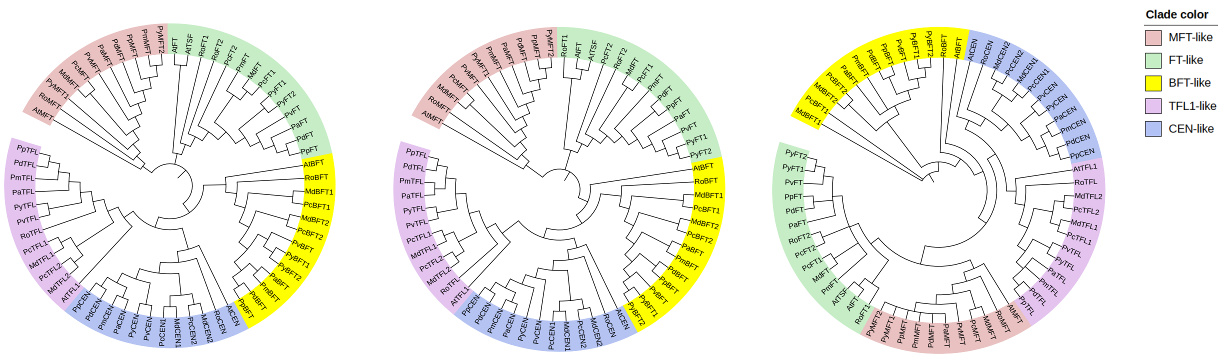

(a) NJ tree **(b)** ML tree **(c)** Bayes tree
