# Supplementary material for: Genome-wide characterization of PEBP family genes in nine Rosaceae tree species and their expression analysis in P. mume
Source: BMC Ecol Evol. 2021 Feb 23;21:32. doi: 10.1186/s12862-021-01762-4 (PMC7901119; doi:10.1186/s12862-021-01762-4)

Figure S2. Exon-intron distributions of *PEBP* family genes from nine *Rosaceae* species and *A. thaliana*.

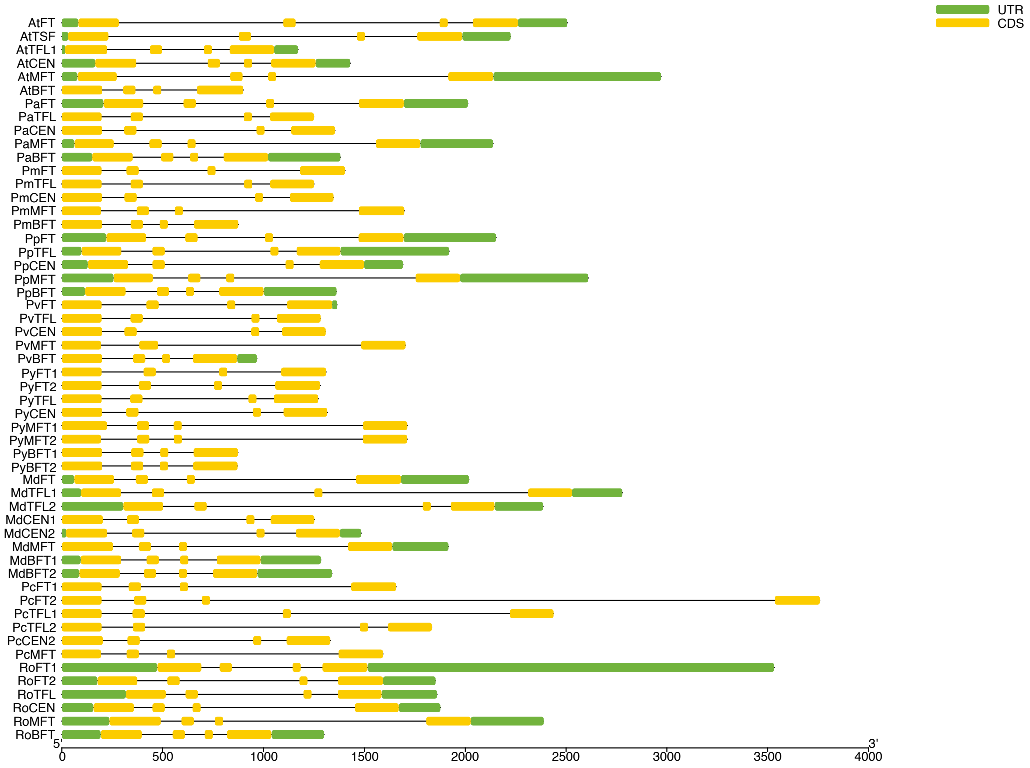

Supplement: Supplementary file 2 — Additional file2: Fig. S2. Exon-intron distributions of PEBP family genes from nine Rosaceae species and A. thaliana. [file 12862_2021_1762_MOESM2_ESM.pdf]
