# Supplementary material for: Genome-wide characterization of PEBP family genes in nine Rosaceae tree species and their expression analysis in P. mume
Source: BMC Ecol Evol. 2021 Feb 23;21:32. doi: 10.1186/s12862-021-01762-4 (PMC7901119; doi:10.1186/s12862-021-01762-4)

Figure S3. Protein sequence alignment of FT and TFL1-like proteins in *Arabidopsis* and nine *Rosaceae* species. Key amino acids responsible for flowering-promoting activity are labeled with red circles; residues at positions critical for 14-3-3 protein binding are labeled with orange circles; and other residues distinguishing FTs from TFLs are labeled with blue circle. The amino acid position is defined as the position in the alignment and not the position in the protein.

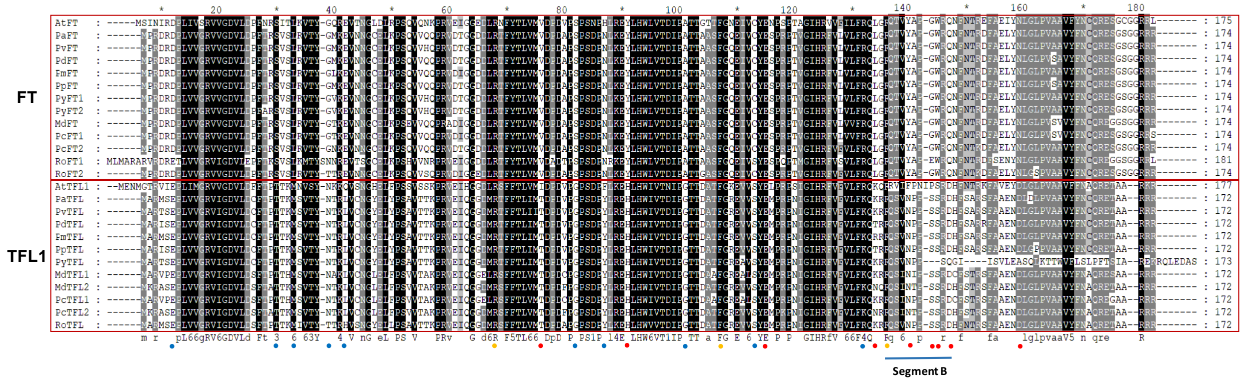

Supplement: Supplementary file 3 — Additional file 3: Fig. S3. Protein sequence alignment of FT and TFL1-like proteins in Arabidopsis and nine Rosaceae species. [file 12862_2021_1762_MOESM3_ESM.pdf]
