# Supplementary material for: Genome-wide characterization of PEBP family genes in nine Rosaceae tree species and their expression analysis in P. mume
Source: BMC Ecol Evol. 2021 Feb 23;21:32. doi: 10.1186/s12862-021-01762-4 (PMC7901119; doi:10.1186/s12862-021-01762-4)

Figure S4. Inter-genomic synteny blocks between species. Comparisons include (a) *A. thaliana* vs *R. occidentalis*, (b) *R. occidentalis* vs *M. domestica*, (c) *M. domestica* vs *P. avium*, (d) *P. avium* vs *P. persica*, (e) *P. persica* vs *P. armeniaca*, and (f) *P. armeniaca* vs *P. mume*.

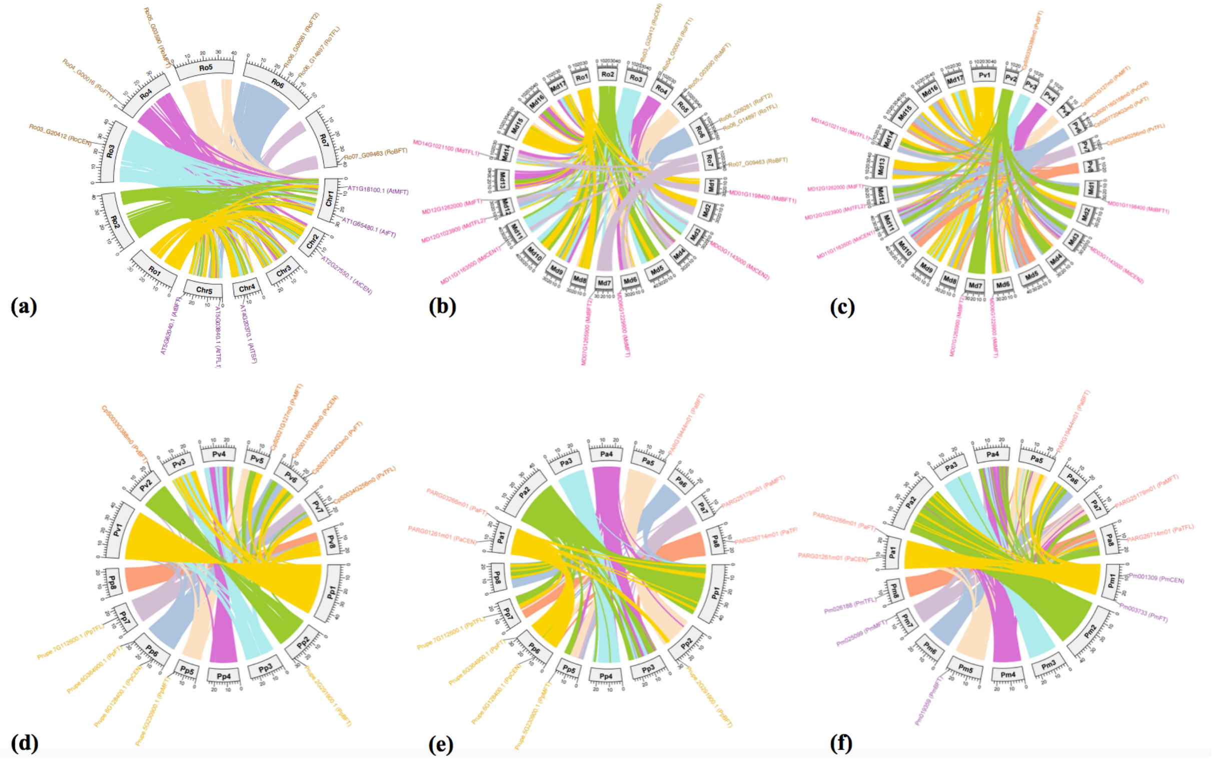

Supplement: Supplementary file 4 — Additional file4: Fig. S4. Inter-genomic synteny blocks between species. [file 12862_2021_1762_MOESM4_ESM.pdf]
