# Supplementary material for: Genome-wide characterization of PEBP family genes in nine Rosaceae tree species and their expression analysis in P. mume
Source: BMC Ecol Evol. 2021 Feb 23;21:32. doi: 10.1186/s12862-021-01762-4 (PMC7901119; doi:10.1186/s12862-021-01762-4)

Figure S5. Distribution and collinearity of *PEBP* family genes within genomes of nine *Rosaceae* species. Species include (a) *M. domestica*, (b) *Pyrus communis*, (c) *R. occidentalis*, (d) *P. armeniaca*, (e) *P. persica*, (f) *P. dulcis*, (g) *P. yedoensis*, (g) *P. mume*, (h) *P. avium*. Syntenic blocks containing *PEBP* gene members were connected with colored lines.

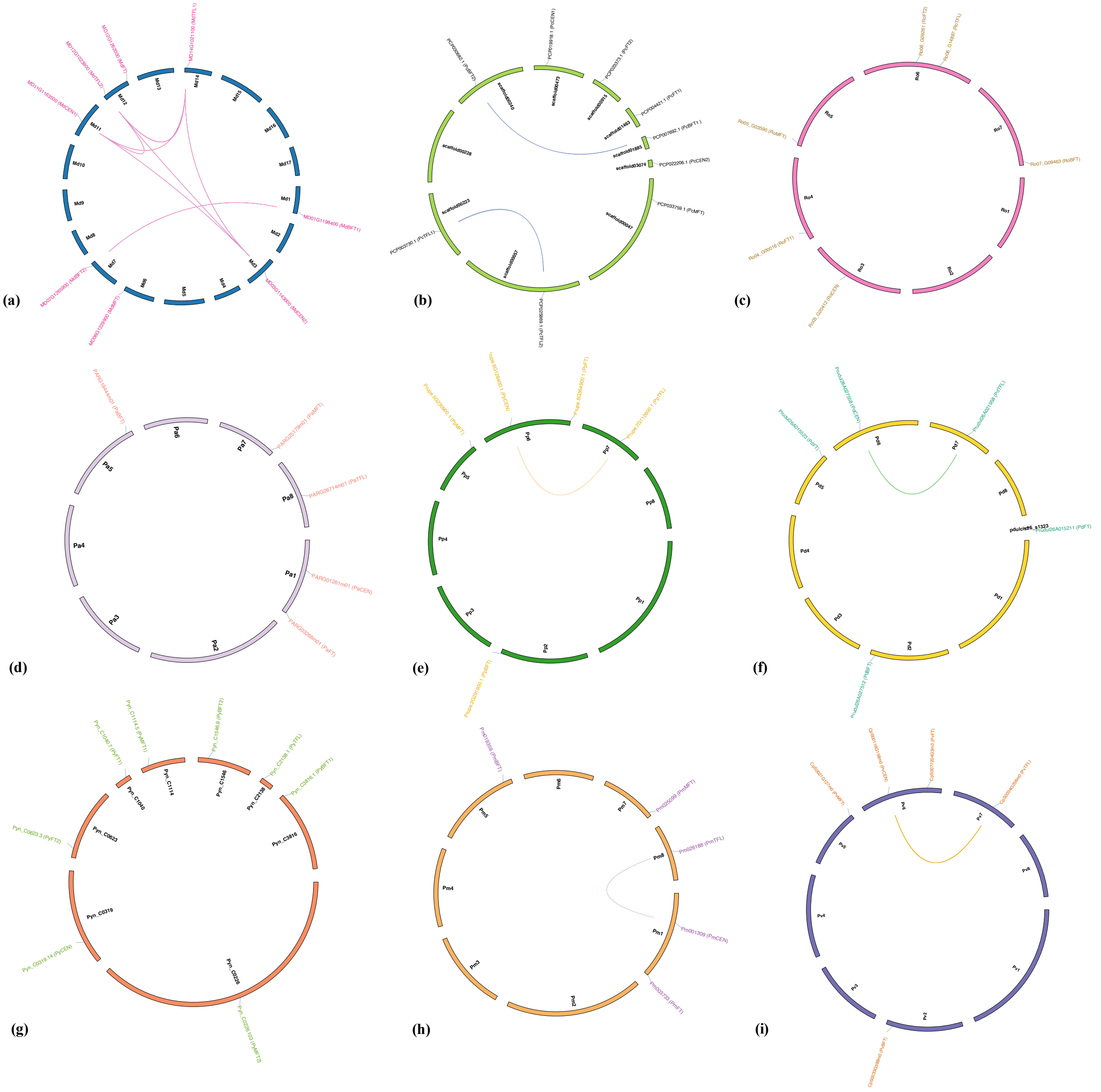

Supplement: Supplementary file 5 — Additional file 5: Fig. S5. Distribution and collinearity of PEBP family genes within genomes of nine Rosaceae species. [file 12862_2021_1762_MOESM5_ESM.pdf]
