# Supplementary material for: Genome-wide characterization of PEBP family genes in nine Rosaceae tree species and their expression analysis in P. mume
Source: BMC Ecol Evol. 2021 Feb 23;21:32. doi: 10.1186/s12862-021-01762-4 (PMC7901119; doi:10.1186/s12862-021-01762-4)

Figure S7. Comparison of gene parameters including the (a) CAI, (b) total GC%, (c) ENC, (d) GC1%, (e) GC2%, (f) GC3% for all *PEBP* genes across nine *Rosaceae* species.

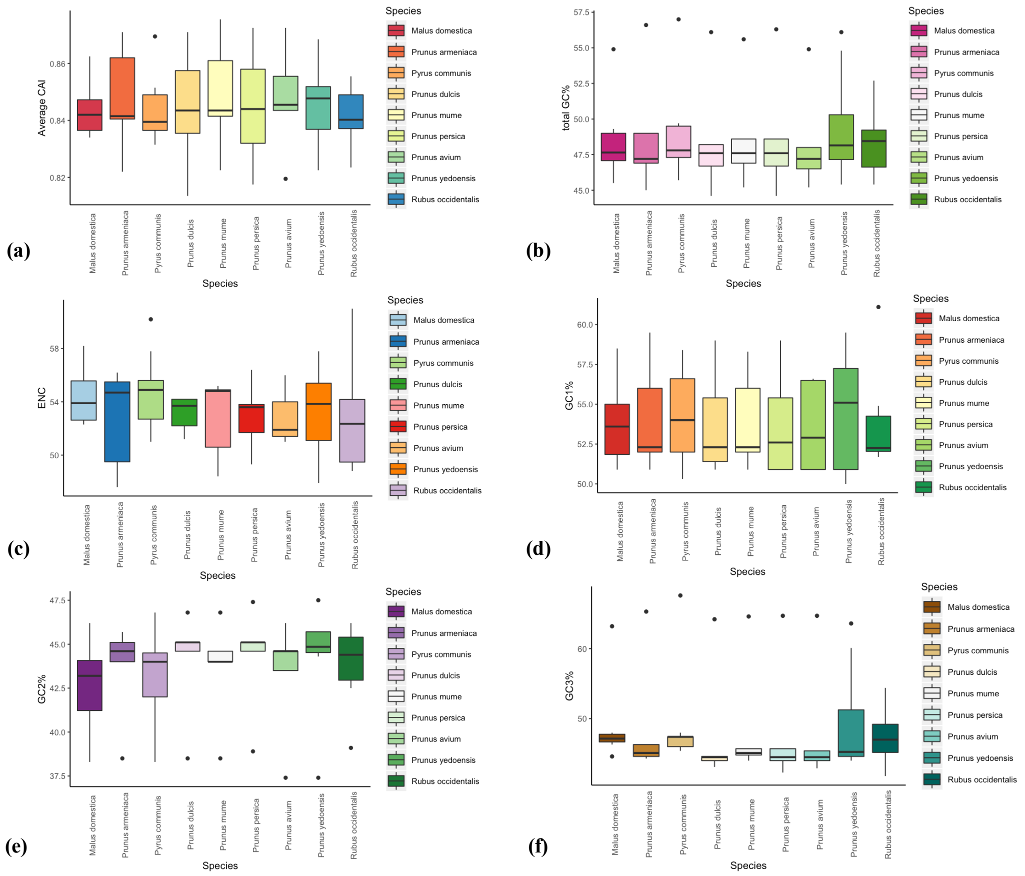

Supplement: Supplementary file 7 — Additional file 7: Fig. S7. Comparison of gene parameters including the (a) CAI, (b) total GC%, (c) ENC, (d) GC1%, (e) GC2%, (f) GC3% for all PEBP genes across nine Rosaceae species. [file 12862_2021_1762_MOESM7_ESM.pdf]
