# Supplementary material for: Genome-wide characterization of PEBP family genes in nine Rosaceae tree species and their expression analysis in P. mume
Source: BMC Ecol Evol. 2021 Feb 23;21:32. doi: 10.1186/s12862-021-01762-4 (PMC7901119; doi:10.1186/s12862-021-01762-4)

Figure S8. Selective pressure analysis of the *BFT, CEN,* and *MFT* lineages and *PEBP* genes as a whole identified strong purifying selection across protein sites. Amino acids colored with yellow/purple indicate sites of positive/purifying selection respectively.

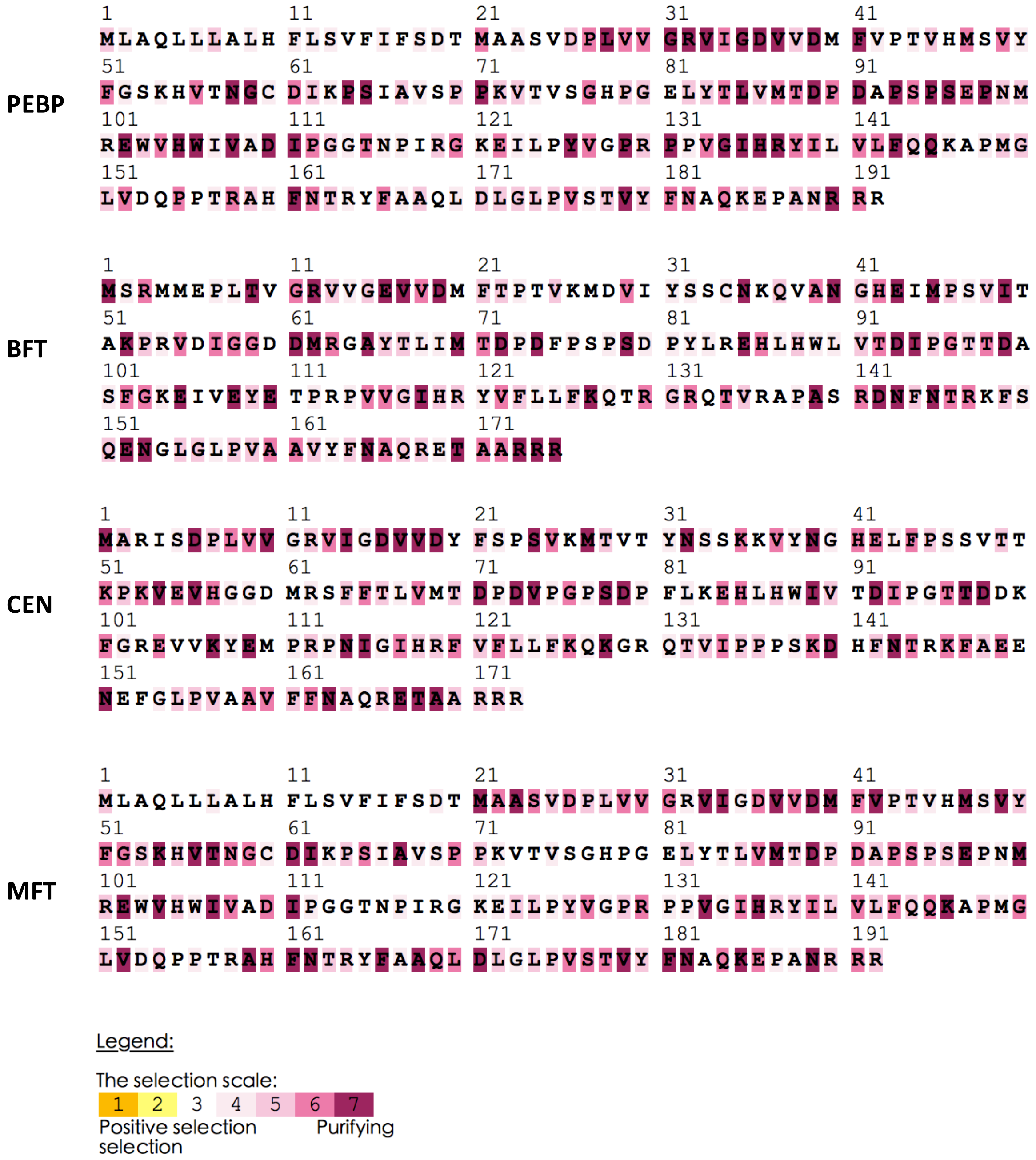

Supplement: Supplementary file 8 — Additional file 8: Fig. S8. Selective pressure analysis of the BFT, CEN, and MFT lineages and PEBP genes as a whole identified strong purifying selection across protein sites. [file 12862_2021_1762_MOESM8_ESM.pdf]
