# Supplementary material for: Genome-wide characterization of PEBP family genes in nine Rosaceae tree species and their expression analysis in P. mume
Source: BMC Ecol Evol. 2021 Feb 23;21:32. doi: 10.1186/s12862-021-01762-4 (PMC7901119; doi:10.1186/s12862-021-01762-4)

Figure S9. Relative expression of *PEBP* family genes in floral bud, leaf, stem, and root tissues of *P. mume*.

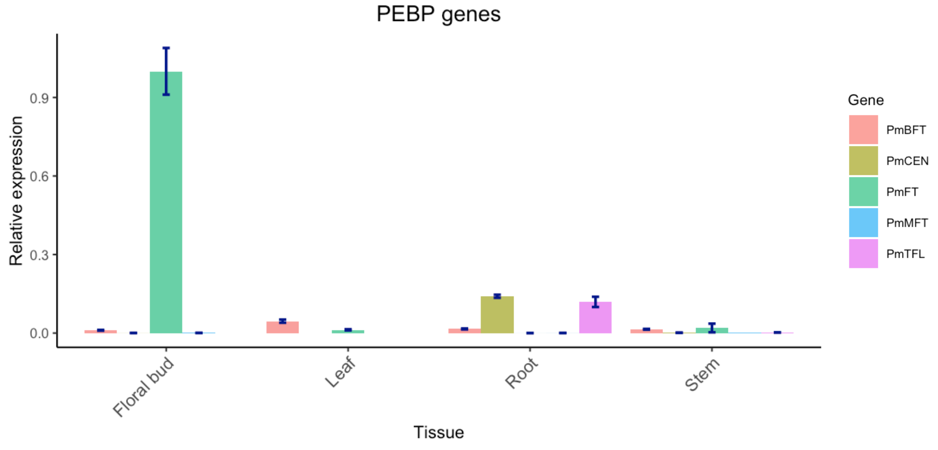

Supplement: Supplementary file 9 — Additional file 9: Fig. S9. Relative expression of PEBP family genes in floral bud, leaf, stem, and root tissues of P. mume. [file 12862_2021_1762_MOESM9_ESM.pdf]
