# Supplementary material for: Genome-wide characterization of PEBP family genes in nine Rosaceae tree species and their expression analysis in P. mume
Source: BMC Ecol Evol. 2021 Feb 23;21:32. doi: 10.1186/s12862-021-01762-4 (PMC7901119; doi:10.1186/s12862-021-01762-4)

Figure S10. Weighted gene co-expression network analysis (WGCNA) during four stages of floral bud blooming in *P. mume*. (a) Hierarchical clustering resulted in 23 modules of co-expressed genes. (b) Module-trait relationship heatmap presenting key modules strongly correlated with traits of interest.

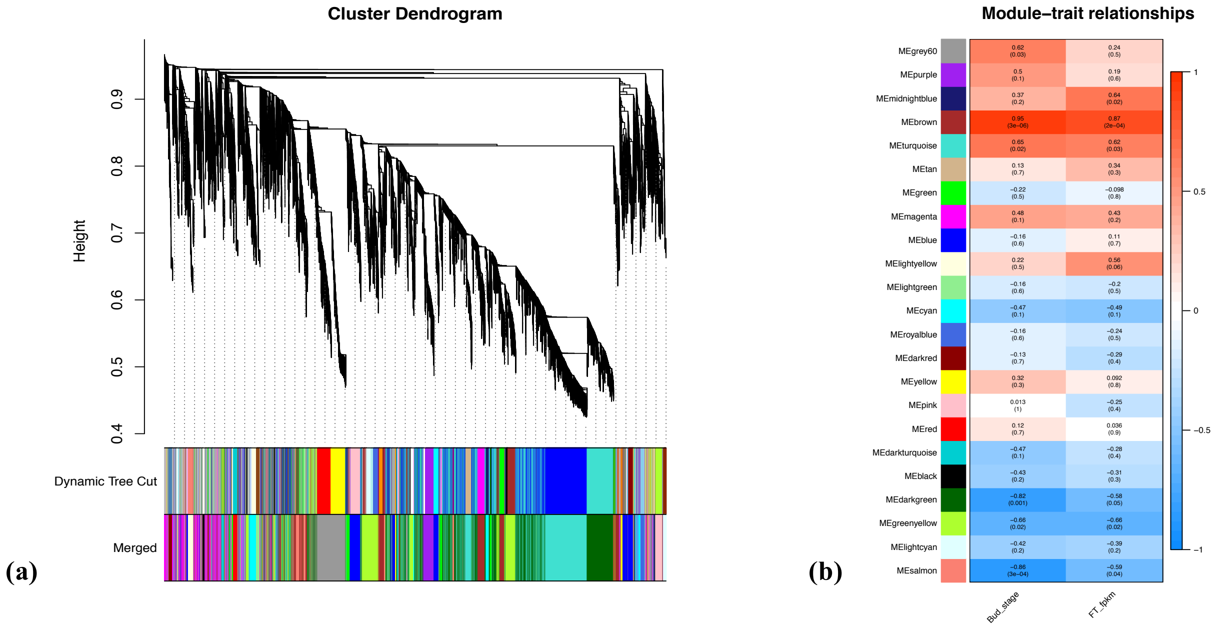

Supplement: Supplementary file 10 — Additional file 10: Fig. S10. Weighted gene co-expression network analysis (WGCNA) during four stages of floral bud blooming in P. mume. [file 12862_2021_1762_MOESM10_ESM.pdf]
