# Supplementary material for: Genome-wide characterization of PEBP family genes in nine Rosaceae tree species and their expression analysis in P. mume
Source: BMC Ecol Evol. 2021 Feb 23;21:32. doi: 10.1186/s12862-021-01762-4 (PMC7901119; doi:10.1186/s12862-021-01762-4)
Supplement: Supplementary file 11 — Additional file 11: TableS1. Numbers of genes originated from different types of duplication events in the genomes of Arabidopsis and seven Rosaceae species. [file 12862_2021_1762_MOESM11_ESM.pdf]

Table S1. Numbers of genes originated from different types of duplication events in the genomes of *Arabidopsis* and seven *Rosaceae* species.

Species	No. genes of duplication origin				
	Singleton	Dispersed	Proximal	Tandem	Segmental/WGD
Arabidopsis thaliana	4246	9035	1081	14204	6820
Malus domestica	4478	9898	3399	3391	23950
Rubus occidentalis	7779	16259	3019	2441	3788
Prunus armeniaca	2561	12297	4217	30232	2789
Prunus persica	3812	7880	2852	29439	3106
Prunus mume	5237	15565	2884	4546	3158
Prunus avium	3138	19047	1135	3713	3942
Prunus dulcis	4548	13535	1983	2989	3987
