# Supplementary material for: Genome-wide characterization of PEBP family genes in nine Rosaceae tree species and their expression analysis in P. mume
Source: BMC Ecol Evol. 2021 Feb 23;21:32. doi: 10.1186/s12862-021-01762-4 (PMC7901119; doi:10.1186/s12862-021-01762-4)
Supplement: Supplementary file 12 — Additional file 12: TableS2. The duplication modes of PEBP genes in the genomes of Arabidopsis and seven Rosaceae species. [file 12862_2021_1762_MOESM12_ESM.pdf]

Table S2. The duplication modes of *PEBP* genes in the genomes of *Arabidopsis* and seven *Rosaceae* species.

Species	No. PEBP genes	Duplication origin				
		Singleton	Dispersed	Proximal	Tandem	Segmental/WGD
Arabidopsis thaliana	6	all	0	0	0	0
Malus domestica	8	*MdFT; MdMFT*	0	0	0	*MdTFL1; MdTFL2; MdCEN1; MdCEN2; MdBFT1; MdBFT2*
Rubus occidentalis	6	all	0	0	0	0
Prunus armeniaca	5	all	0	0	0	0
Prunus persica	5	*PpFT; PpMFT; PpBFT*	0	0	0	*PpTFL; PpCEN*
Prunus mume	5	*PmFT; PmMFT; PmBFT*	0	0	0	*PmTFL; PmCEN*
Prunus avium	5	*PvFT; PvMFT; PvBFT*	0	0	0	*PvTFL; PvCEN*
Prunus dulcis	5	*PdFT; PdMFT; PdBFT*	0	0	0	*PdTFL; PdCEN*
