# Supplementary material for: Genome-wide characterization of PEBP family genes in nine Rosaceae tree species and their expression analysis in P. mume
Source: BMC Ecol Evol. 2021 Feb 23;21:32. doi: 10.1186/s12862-021-01762-4 (PMC7901119; doi:10.1186/s12862-021-01762-4)
Supplement: Supplementary file 13 — Additional file 13: TableS3. Pearson correlation between gene parameters estimated for 56 Rosaceae PEBP genes. [file 12862_2021_1762_MOESM13_ESM.pdf]

Table S3. Pearson correlation between gene parameters estimated for 56 *Rosaceae PEBP* genes. Correlation coefficients with significant adjusted p-value <0.05 were shown in bold font. Positive correlation coefficients were colored in red and negative correlations colored in blue.

	Length	CAI	totalGC%	GC1%	GC2%	GC3%	ENC
Length		-0.10	0.19	0.32	-0.03	0.16	0.19
CAI	-0.10		**0.56**	0.09	0.16	**0.72**	**-0.62**
totalGC%	0.19	**0.56**		**0.80**	**0.45**	**0.93**	-0.34
GC1%	0.32	0.09	**0.80**		0.17	**0.67**	0.09
GC2%	-0.03	0.16	**0.45**	0.17		0.18	-0.26
GC3%	0.16	**0.72**	**0.93**	**0.67**	0.18		**-0.43**
ENC	0.19	**-0.62**	-0.34	0.09	-0.26	**-0.43**	
