# Supplementary material for: Genome-wide characterization of PEBP family genes in nine Rosaceae tree species and their expression analysis in P. mume
Source: BMC Ecol Evol. 2021 Feb 23;21:32. doi: 10.1186/s12862-021-01762-4 (PMC7901119; doi:10.1186/s12862-021-01762-4)
Supplement: Supplementary file 14 — Additional file 14: TableS4. Parameter estimates and likelihood values for branch models among PEBP lineages. [file 12862_2021_1762_MOESM14_ESM.pdf]

Table S4. Parameter estimates and likelihood values for branch models among *PEBP* lineages. Significant chi-square comparisons were indicated with * (pLRT<0.05), ** (pLRT<0.01), *** (pLRT<0.001).

Model Type	Branch Model	Fore-ground branch	LnL	Estimate of parameters	Model comparison	pLRT
0	Fixed	-	-4634.412778	ω=1.15119		
1	Free-Ratio		-4578.273183	-	Model 1 vs Model 0	0.370
2	1	FT	-4634.024489	ω_1=1.12589 ω_2=1.65765	Model 2 vs Model 1	0.364
2	2	TFL1	-4630.306725	ω_1=1.10494 ω_2=999.000	Model 2 vs Model 1	0.562
2	3	CEN	-4634.056295	ω_1=1.17099 ω_2=0.78996	Model 2 vs Model 1	0.362
2	4	BFT	-4634.306541	ω_1=1.16330 ω_2=0.91937	Model 2 vs Model 1	0.350
2	5	(FT, BFT, TFL1, CEN)	-4634.316158	ω_1=1.16488 ω_2=0.99487	Model 2 vs Model 1	P<0.001 ***
