# Supplementary material for: Genome-wide characterization of PEBP family genes in nine Rosaceae tree species and their expression analysis in P. mume
Source: BMC Ecol Evol. 2021 Feb 23;21:32. doi: 10.1186/s12862-021-01762-4 (PMC7901119; doi:10.1186/s12862-021-01762-4)
Supplement: Supplementary file 15 — Additional file 15: TableS5. Parameter estimates and likelihood values for site models across PEBP protein sites. [file 12862_2021_1762_MOESM15_ESM.pdf]

Table S5. Parameter estimates and likelihood values for site models across PEBP protein sites. Significant chi-square comparisons were indicated with * (pLRT<0.05), ** (pLRT<0.01), *** (pLRT<0.001). Positive selected sites with its probability were detected with Bayes Empirical Bayes analysis.

Nsite model	LnL	Estimate of parameters	pLRT	Selected sites
M0: one ratio	-4634.412	ω=1.15119		
M1: neutral	-4593.888	p_1=0.23775, p_2=0.76225 ω_1=0.14634, ω_2=1.00000		
M2: selection	-4575.12 ***	p_1=0.19566, p_2=0.42586, p_3=0.37847 ω_1=0.14968, ω_2=1.00000, ω_3=2.10351	7.07E-09 (M1 vs M2)	37,66,93,100,102,104,117,123
M7: beta (neutral)	-4595.246	p=0.10000, 0.10000, 0.10000, 0.10000, 0.10000, 0.10000, 0.10000, 0.10000, 0.10000, 0.10000 ω= 0.00000, 0.21609, 0.99957, 1.00000, 1.00000, 1.00000, 1.00000, 1.00000, 1.00000, 1.00000		
M8: beta (selection)	-4574.797 ***	p=0.05577, 0.05577, 0.05577, 0.05577, 0.05577, 0.05577, 0.05577, 0.05577, 0.05577, 0.05577, 0.44227 ω=0.01239, 0.11457, 0.29660, 0.50895, 0.70252, 0.84742, 0.93706, 0.98142, 0.99715, 0.99995, 1.97271	1.32E-09 (M7 vs M8)	13,19,21,34,37,61,66,69,73,76,83,89,90,91,93,94,96,100,102,104,105,117,121,122,123
