# Supplementary material for: Genome-wide characterization of PEBP family genes in nine Rosaceae tree species and their expression analysis in P. mume
Source: BMC Ecol Evol. 2021 Feb 23;21:32. doi: 10.1186/s12862-021-01762-4 (PMC7901119; doi:10.1186/s12862-021-01762-4)
Supplement: Supplementary file 16 — Additional file 16: TableS6. Candidate genes co-expressed with PmFTduring dormancy release in P. mume. [file 12862_2021_1762_MOESM16_ESM.pdf]

Table S6. Candidate genes co-expressed with *PmFT* during dormancy release in *P. mume*.

ID	Name	Arabidopsis ortholog	Gene description
Pm000015	Pm000015	AT4G02620	ATP synthase (F/14-kDa) subunit
Pm000354	Pm000354	-	-
Pm000576	Pm000576	AT2G29970	SMXL7, SMAX1-LIKE 7
Pm001840	Pm001840	AT2G21950	SKIP6, SKP1 INTERACTING PARTNER 6
Pm002166	PmSVP	AT2G22540	SHORT VEGETATIVE PHASE, SVP
Pm002433	Pm002433	AT2G23140	PUB4, ubiquitin-protein ligase
Pm003287	Pm003287	AT1G09020	CBS domain containing membrane protein
Pm003731	Pm003731	AT5G36700	Haloacid dehalogenase-like hydrolase
Pm004212	Pm004212	AT4G09900	MES12, METHYL ESTERASE 12
Pm004415	PmDAM6	-	DAM6, Dormancy-associated MADS-box transcription factor 6
Pm004416	PmDAM5	-	DAM5, Dormancy-associated MADS-box transcription factor 5
Pm004417	PmDAM4	-	DAM4, Dormancy-associated MADS-box transcription factor 4
Pm004420	PmDAM1	-	DAM1, Dormancy-associated MADS-box transcription factor 1
Pm004718	Pm004718	AT5G20240	PI, PISTILLATA
Pm004871	Pm004871	-	-
Pm005002	Pm005002	AT4G32530	ATPase
Pm006700	Pm006700	AT1G60590	Pectin lyase-like superfamily protein
Pm006712	Pm006712	AT1G60810	ATP Citrate lyase
Pm007747	Pm007747	AT4G14040	selenium-binding protein 2
Pm008367	Pm008367	AT2G18790	Phytochrome B
Pm008666	Pm008666	AT4G30310	FGGY family of carbohydrate kinase
Pm008685	Pm008685	AT5G57630	Protein kinase domain
Pm008925	Pm008925	AT1G14650	SWAP (Suppressor-of-White-APricot)/surp domain-containing protein
Pm010392	Pm010392	AT2G34470	UREG, UREASE ACCESSORY PROTEIN G
Pm012110	Pm012110	AT1G21200	Myb/SANT-like DNA-binding domain
Pm012952	Pm012952	AT2G31400	pentatricopeptide-repeat protein
Pm013633		AT4G14770	TESMIN/TSO1-like CXC 2
Pm013793	Pm013793	AT1G80860	defective in cullin neddylation protein (DUF298)
Pm014348		AT5G37790	Protein kinase domain
Pm014730	Pm014730	AT1G48320	Thioesterase superfamily protein
Pm014767	Pm014767	AT2G30860	GSTF9, GLUTATHIONE S-TRANSFERASE PHI 9

Pm015446	PmCOL	AT3G02380	COL, CONSTANS-like 2
Pm016910	Pm016910	AT2G26230	UOX, URATE OXIDASE
Pm017632	Pm017632	AT3G22320	RNA polymerase Rpb5
Pm018089	PmSOC1	AT2G45660	SOC1
Pm018577	Pm018577	AT1G07930	GTP binding Elongation factor Tu family protein
Pm018676	Pm018676	AT2G39830	LRD3, LATERAL ROOT DEVELOPMENT 3
Pm019359	PmCEN	AT2G27550	CEN, Centroradialis
Pm020062	Pm020062	AT2G40940	ERS, ETHYLENE RESPONSE SENSOR
Pm020108	Pm020108	AT3G62580	Late embryogenesis abundant protein (LEA) family protein
Pm021191	Pm021191	AT4G35220	cyclase family protein
Pm021763	PmCIB1	AT4G34530	CIB1, CRYPTOCHROME-INTERACTING BASIC-HELIX-LOOP-HELIX 1
Pm021986	Pm021986	AT5G64380	Inositol monophosphatase family protein
Pm022471	PmGI	AT1G22770	GI, GIGANTEA
Pm023206	Pm023206	AT4G17910	GPI-anchored wall transfer protein 1
Pm023520	Pm023520	AT1G62500	Bifunctional inhibitor/lipid-transfer protein
Pm023787	Pm024079	AT1G12780	UDP-glucose 4-epimerase
Pm024610	PmLFY	AT5G61850	LFY, LEAFY
Pm025571	Pm025571	AT4G24380	Serine hydrolase (FSH1)
Pm025831	Pm025831	AT3G57790	Glycosyl hydrolases family 28
Pm026199	Pm026199	AT2G36310	NSH1, NUCLEOSIDE HYDROLASE 1
Pm026270	Pm026270	AT5G03790	homeobox associated leucine zipper
Pm027037	Pm027037	AT5G12080	mechanosensitive channel of small conductance-like 10
Pm027079	Pm027079	AT3G45310	oryzain gamma chain precursor
Pm027498	Pm027283	AT4G29260	HAD superfamily phosphatase
Pm028503	Pm028503	AT4G01410	Late embryogenesis abundant protein
Pm028542	Pm028542	AT5G63000	Mitochondrial import inner membrane translocase
Pm028731	PmLHY1	AT1G01060	LHY1, LATE ELONGATED HYPOCOTYL 1
Pm030191	Pm030191	AT1G73920	alpha/beta-Hydrolases superfamily protein
Pm030594	PmAP1	AT1G69120	AP1, APETALA1
Pm030895	Pm030895	AT1G73020	alpha/beta-Hydrolases superfamily protein
