# Supplementary material for: Genome-wide characterization of PEBP family genes in nine Rosaceae tree species and their expression analysis in P. mume
Source: BMC Ecol Evol. 2021 Feb 23;21:32. doi: 10.1186/s12862-021-01762-4 (PMC7901119; doi:10.1186/s12862-021-01762-4)
Supplement: Supplementary file 17 — Additional file 17: TableS7. Primers used in qRT-PCR analysis for PEBPfamily genes in P. mume. [file 12862_2021_1762_MOESM17_ESM.pdf]

Table S7. Primers used for qRT-PCR analysis for *PEBP* family genes in *P. mume*.

Gene	Primer name	Sequence
PmBFT	BFT-F	GACAAGTTGCCAATGGACATG
	BFT-R	CTCTCAAGTAAGGATCACTGGG
PmCEN	CEN-F	CTTCCTCACTAACCCTCAAGC
	CEN-R	TGTGGTATCAGTTGTGCCTG
PmFT	FT-F	GCTCAAACCTTCCCAAGTTG
	FT-R1	TGCTGGTATATCCGTAACCAACC
PmMFT	MFT-F	GTCCAAGCATGTCACCAATG
	MFT-R	TGTCAGCCACGATCCAATG
PmTFL	TFL1-F	CTGTCACCACCAAACCTAGAG
	TFL1-R	TCACCACCTCTCTTCCAAATG
