# Supplementary material for: Genome-wide characterization of PEBP family genes in nine Rosaceae tree species and their expression analysis in P. mume
Source: BMC Ecol Evol. 2021 Feb 23;21:32. doi: 10.1186/s12862-021-01762-4 (PMC7901119; doi:10.1186/s12862-021-01762-4)
Supplement: Supplementary file 18 — Additional file 18: TableS8. Primers used in qRT-PCR analysis for FTco-expressed genes in P. mume. [file 12862_2021_1762_MOESM18_ESM.pdf]

Table S8. Primers used for qRT-PCR analysis of putative *FT* co-expressed genes in *P. mume*.

Gene	Primer name	Sequence
PmPP2A	PP2A-F	AGGGTTCGGCTCGCAATAATAGA
	PP2A-R	TGTTAGCAGCAGCATCACGAAT
PmDAM1	DAM1-F	AGTATGAAGGATGTTATTCAA
	DAM1-R	CTTAAGTTCCTTGCTCAATCT
PmDAM4	DAM4-F	ACCCTTGTCCGTGTGATGGAA
	DAM4-R	ATCACCATCTGATTGTTGCCT
PmDAM5	DAM5-F	AGGCTGAATAATAATATTGAA
	DAM5-R	TTAACGCCCCAGTTTGAGAGA
PmDAM6	DAM6-F	AACCAACAACCAGTTAAGGCATA
	DAM6-R	CAATTACGGCAGATTCAGATGA
Pm004212	Pm004212-F	ATTGTCTCAGAGGTTTGACGG
	Pm004212-R	CTCCAAACCCTTCTCCATGTAC
Pm004718	Pm004718-F	CAAGATTGAGATTAAGAGGATTG
	Pm004718-R	AGAACAGTGATTTCCTTGGC
Pm008367	Pm008367-F	AGATAGTTTGGGTGATGCTGG
	Pm008367-R	TCTCTTTTGCAGTGTGGGATC
PmCOL	COL-F	TCCACTCTGCCAATCCGTTG
	COL-R	AGCAGACCCCACCTCTATCC
PmSOC1	SOC1-F	GTTCTTTGTGATGCTGAGGTTG
	SOC1-R	GCTGCATATTTTGGTCAGTGG
PmCIB1	CIB1-F	GAAGAGGAAGGCTGATAAGGTG
	CIB1-R	GTTTGTGTTCTTGGTGCTGG
Pm025831	Pm025831-F	ATCCAATCTGCCATCGACAG
	Pm025831-R	TTGTTCTGCTCTTTCGGGTAG
Pm026270	Pm026270-F	CACCGGGTACACTGAAATCTC
	Pm026270-R	ACTCTTGCACCTGATGTTGG
PmAP1	AP1-F	AGTACGCCACGGATTCATG
	AP1-R	CTTCTCCCAAATAGTGCCTCTG
